# Prevention of allergy by virus‐like nanoparticles (VNP) delivering shielded versions of major allergens in a humanized murine allergy model

**DOI:** 10.1111/all.13573

**Published:** 2018-11-05

**Authors:** Bernhard Kratzer, Cordula Köhler, Sandra Hofer, Ursula Smole, Doris Trapin, Jagoba Iturri, Dietmar Pum, Philip Kienzl, Adelheid Elbe‐Bürger, Pia Gattinger, Irene Mittermann, Birgit Linhart, Gabriele Gadermaier, Beatrice Jahn‐Schmid, Alina Neunkirchner, Rudolf Valenta, Winfried F. Pickl

**Affiliations:** ^1^ Institute of Immunology Center for Pathophysiology, Infectiology and Immunology Medical University of Vienna Vienna Austria; ^2^ Department of Nanobiotechnology Institute for Biophysics University of Natural Resources and Life Sciences Vienna Vienna Austria; ^3^ Department of Dermatology Division of Immunology, Allergy and Infectious Diseases Medical University of Vienna Vienna Austria; ^4^ Department of Pathophysiology and Allergy Research Center for Pathophysiology, Infectiology and Immunology Medical University of Vienna Vienna Austria; ^5^ Division of Allergy and Immunology Department of Biosciences University of Salzburg Salzburg Austria

**Keywords:** allergy prevention, mugwort pollen allergy, Treg cells, virus‐like nanoparticles, prevention, lung APC

## Abstract

**Background:**

In high‐risk populations, allergen‐specific prophylaxis could protect from sensitization and subsequent development of allergic disease. However, such treatment might itself induce sensitization and allergies, thus requiring hypoallergenic vaccine formulations. We here characterized the preventive potential of virus‐like nanoparticles (VNP) expressing surface‐exposed or shielded allergens.

**Methods:**

Full‐length major mugwort pollen allergen Art v 1 was selectively targeted either to the surface or to the inner side of the lipid bilayer envelope of VNP. Upon biochemical and immunological analysis, their preventive potential was determined in a humanized mouse model of mugwort pollen allergy.

**Results:**

Virus‐like nanoparticles expressing shielded version of Art v 1, in contrast to those expressing surface‐exposed Art v 1, were hypoallergenic as they hardly induced degranulation of rat basophil leukemia cells sensitized with Art v 1‐specific mouse or human IgE. Both VNP versions induced proliferation and cytokine production of allergen‐specific T cells *in vitro*. Upon intranasal application in mice, VNP expressing surface‐exposed but not shielded allergen induced allergen‐specific antibodies, including IgE. Notably, preventive treatment with VNP expressing shielded allergen‐protected mice from subsequent sensitization with mugwort pollen extract. Protection was associated with a Th1/Treg‐dominated cytokine response, increased Foxp3^+^ Treg numbers in lungs, and reduced lung resistance when compared to mice treated with empty particles.

**Conclusion:**

Virus‐like nanoparticles represent a novel and versatile platform for the *in vivo* delivery of allergens to selectively target T cells and prevent allergies without inducing allergic reactions or allergic sensitization.

**Abbreviations**
AHR: airway hyper‐reactivity
AIT: allergen‐specific immunotherapy
APC: antigen‐presenting cell
BMDC: bone marrow‐derived dendritic cells
DC: dendritic cells
DLS: dynamic light scattering
DRM: detergent‐resistant membrane fraction
DSM: detergent‐soluble membrane fraction
Foxp3: forkhead box protein 3
FP: fusion protein
GPI: glycosylphosphatidylinositol
i.n.: intranasally
IB: immunoblot
MA: MoMLV matrix protein
MoMLV: moloney murine leukemia virus
OGP: MoMLV Gag‐Pol
pTreg: peripheral Treg
RBL: rat basophil leukemia cells
TCR: T cell receptor
Teff: T effector cells
Treg: regulatory T cell
VNP: virus‐like nanoparticles

**Highlights**

- Full‐length allergens can be specifically targeted to the inner leaflet of MoMLV virus‐like nanoparticles by fusion to the viral matrix protein MAp15 and thus remain shielded from IgE recognition
- Shielded allergens are hypoallergenic, do not activate, but become engulfed by distinct subsets of antigen‐presenting cells and stimulate allergen‐specific T cells for proliferation and factor production
- Prophylactic application of VNP in a humanized mouse model of mugwort allergy protects from sensitization, elevates lung‐resident Foxp3^+^CD4^+^ Treg while suppressing Th2 cytokine production and thereby maintains normal lung function

## 1 INTRODUCTION

More than 25% of the population in Western countries is affected by allergies. Currently available options to manage allergic diseases include allergen avoidance, symptomatic pharmacotherapy,^1^ or in severe cases application of biologics targeting IgE or inflammatory mediators of adaptive or innate immune cells.^2^, ^3^, ^4^, ^5^ while the only disease‐modifying treatment available so far is allergen‐specific immunotherapy with allergen extracts (AIT).^6^, ^7^ However, the poor quality of natural allergen extracts is a major limitation for the development of AIT.^8^, ^9^ Another challenge of AIT is that it can induce allergic side effects which can be potentially life‐threatening.^10^ To overcome the disadvantages of allergen extract‐based AIT, improved forms of molecular allergy vaccines are being developed and clinically evaluated.^11^, ^12^, ^13^, ^14^, ^15^

Therefore, prevention by specific approaches has become an attractive strategy in allergy, especially in high‐risk populations.^11^, ^16^, ^17^ Today, such high‐risk populations can be reliably identified by component‐resolved molecular allergy diagnosis.^18^, ^19^ The presence and degree of sensitization against marker allergens early on in life (ie, at 4 years of age) serve as highly significant prediction markers for manifest disease later in life (ie, at 8 and 16 years of age)^18^ and pave the way for preventive interventions such as those based on virus‐like nanoparticles (VNP).

The principle of linking allergens to particles has been already suggested as possible AIT strategy. This approach can be used to reduce the allergenic activity of allergens and to enhance their immunogenicity.^20^, ^21^ Likewise, VNP may be used as allergen carriers.^22^, ^23^ They are noninfectious enveloped particles that are inducible in mammalian cells by the expression of viral structural proteins (ie, Gag) in the absence of viral nucleic acids or envelope proteins. By rational incorporation^22^ of membrane‐bound cytokines,^24^, ^25^, ^26^, ^27^ cytokine receptors,^25^ growth factors,^24^ fluorescent proteins,^22^, ^25^, ^27^ MHC, costimulatory and also adhesion molecules,^28^, ^29^ VNP have been demonstrated to sustain stimulatory but also inhibitory immune responses and, as fluorescent versions, can also be used to trace, for example, subtle receptor‐ligand interactions by flow cytometry. However, so far, no in‐depth analysis has been performed to investigate how the mode of linking of allergens to VNP may affect their allergenic and tolerogenic properties and if such a strategy can be used to foster T cell regulation. Clinically effective AIT is associated with an increase in allergen‐specific pTreg numbers in already allergic individuals.^30^, ^31^, ^32^ Similarly, increased numbers of pTregs might also reduce the likelihood of (further) sensitization in individuals at risk to develop manifest allergies.^32^, ^33^, ^34^

To study particle‐based allergen delivery, its impact on pTreg generation, and induction of allergen‐specific tolerance, we devised VNP expressing the major mugwort pollen allergen Art v 1 either on the VNP surface (as glycophosphatidylinositol (GPI) fusions) or shielded by the VNP lipid envelope (as viral matrix protein (MA) fusions) and compared the impact of their mucosal application (intranasally (i.n.)) with that of pollen extracts on subsequent allergen exposure.

Moreover, we determined the basophil activation and *in vivo*‐sensitizing capabilities of allergen‐specific VNP, taking advantage of a recently established, human‐relevant TCR/DR1 transgenic mouse model.^35^ These transgenic mice express a human Art v 1_25‐36_‐specific αβTCR and HLA‐DR1 and show hallmarks of allergic disease such as lung inflammation, airway hyper‐responsiveness (AHR), and allergen‐specific IgE upon natural i.n. exposure of mucosal surfaces to aqueous allergen extract in the absence of systemic priming or adjuvants. In addition, we tested whether prophylactic application of allergen‐specific VNP would mitigate allergic sensitization and AHR with allergen extract and we sought to identify the responsible mechanisms.

## 2 MATERIALS AND METHODS

### 2.1 Production of VNP

HEK‐293T (3 × 10^6^) cells were seeded onto 150‐mm culture dishes, transfected the day after with 30 μg of MoMLV original *gag‐pol* (OGP) plasmid and 60 μg of the expression respective plasmids pEAK12::MA::Art v 1, pEAK12:Art v 1::GPI, or empty vector. VNP‐containing supernatants were harvested after 72 hours, filtered (0.45 μm, Millipore, Billerica, MA), concentrated (Centricon Plus‐70, Merck Millipore Ltd., Tullagreen, Ireland), and followed by concentration using a SW41 Ti rotor (1 × 10^5^ g, 1 hour, Beckman‐Optima LE‐80K, Beckman Instruments, Palo Alto, CA).^26^ Protein concentrations of PBS‐washed VNP preparations were determined (Micro BCA, Thermo Fisher, Waltham, MA) and adjusted. VNP were stored at 4°C until use for up to 4 weeks, without alteration of biological activity.

### 2.2 Mice

Age‐matched, female (6‐10 weeks old), homozygous B57BL/6 mice co‐expressing an Art v 1_25‐36_‐specific TCR and HLA‐DRA*01/‐DRB1*01 (HLA‐DR1) heterodimers were used for experiments^35^ according to FELASA 2014 recommendations^36^ and approval by the Ethics Committee of the Medical University of Vienna, No.BMWFW‐66.009/0161‐WF/V/3b/2016.

### 2.3 Statistical analyses

Groups with similar variance were compared using parametric tests (Student's *t* test or one‐way ANOVA) followed by correction of alpha (Tukey or Holms‐Sidak) using GraphPad 6.0 (GraphPad Software Inc., La Jolla, CA). Otherwise, the Mann‐Whitney *U* test or the Kruskal‐Wallis test was performed, followed by Dunn's multiple comparison testing. ns, not significant; *P < 0.05; **P < 0.01; ***P < 0.001.

Further experimental details are provided in the Materials and Methods section in this article's Appendix S1. All antibodies used within this study are listed in Table S1.

## 3 RESULTS

### 3.1 Creation of cell surface‐anchored and shielded versions of allergens on VNP

**Figure 1:**
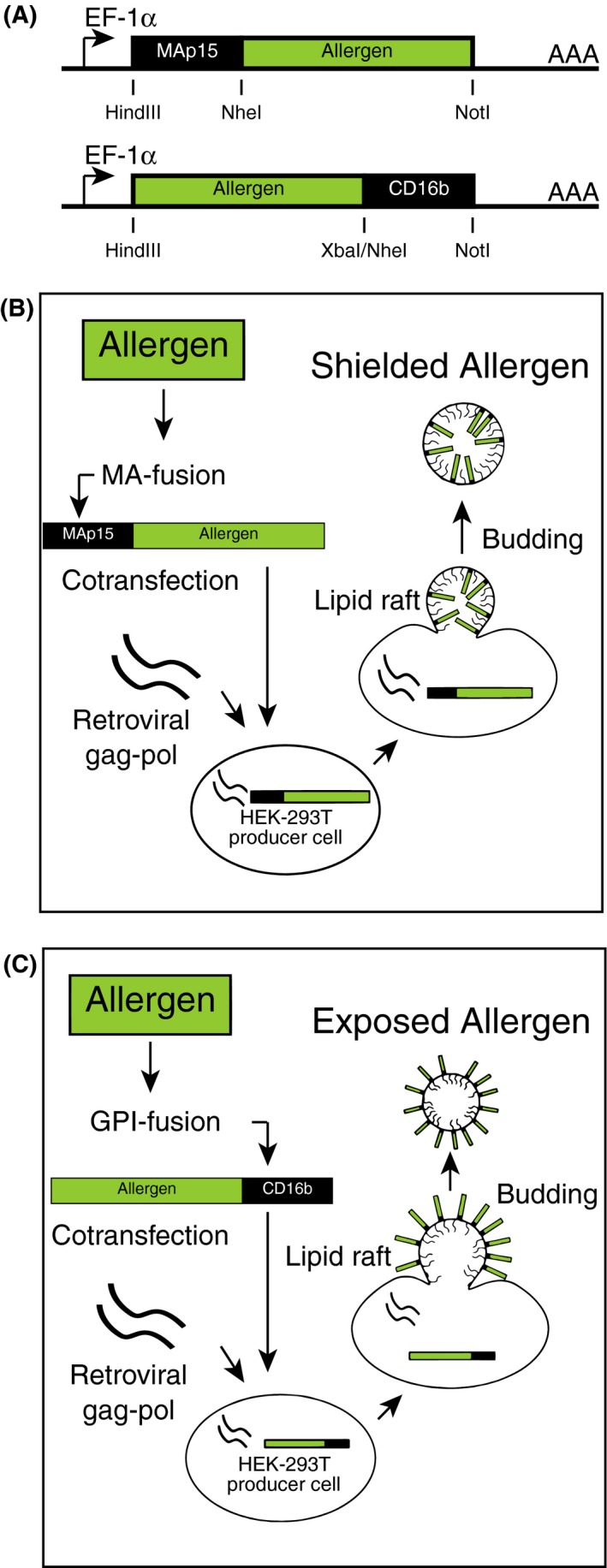
Scheme for the creation of allergen‐fusion proteins (FP) and the production of surface‐expressed and shielded versions of allergen‐expressing virus‐like nanoparticles (VNP). A, MAp15(MA::)FPs were created by inserting Nhe I and Not I‐flanked, codon‐optimized versions of Art v 1 or Bet v 1 into the pEAK12 expression vector containing the Hind III and Nhe I‐tagged MoMLV matrix (MA, p15) DNA sequence.^25^ Glycosylphosphatidylinositol (GPI) FPs were generated by inserting the Hind III and Xba I‐flanked Art v 1 or Bet v 1 sequences generated by PCR amplification into the pEAK12 vector containing the Nhe I and Not I‐flanked minimal GPI anchor acceptor sequence of CD16b.^24^ B and C, Shown are VNP production schemes for shielded (MA::allergen FP) and surface‐exposed (allergen::GPI FP) allergens. Expression of retroviral (MoMLV) *gag‐pol* leads to the formation and budding of VNP from lipid raft‐enriched regions of the plasma membrane of producer cells. MA::allergen FP are targeted to the inner side (B) while allergen::GPI FP become targeted to the surface of the lipid bilayer envelope (C) of nascent VNP due to the differential posttranslational lipid modification of their respective fusion partners

**Figure 2:**
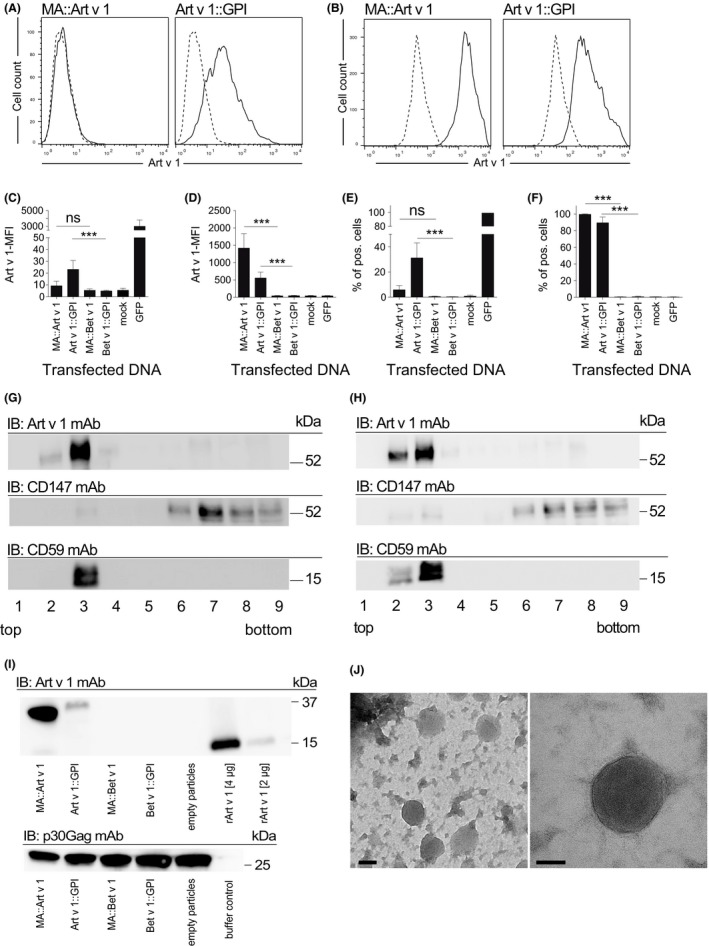
Expression of allergen‐fusion proteins in producer cells and virus‐like nanoparticles (VNP). Flow cytometry analyses of Art v 1 upon A, surface or B, intracellular staining of HEK‐293T cells transiently transfected with MA::Art v 1 or Art v 1:: glycosylphosphatidylinositol (GPI) (solid lines), or negative control plasmid (dashed line). C and D, Shown is the mean fluorescence intensity (MFI), or E and F, the percentage of HEK‐293T cells expressing allergen FP or transfected with control plasmids (mock and GFP), upon surface (C and E) or intracellular (D and F) staining with the anti‐Art v 1 clone 5 mAb (mean ± SD).^38^ Isopycnic sucrose gradients of G, MA::Art v 1 or H, Art v 1::GPI‐transfected HEK‐293T cell lysates immunoblotted (IB) with anti‐Art v 1, CD59, or CD147 mAb. Fractions are numbered from top 1 (5%) to bottom 9 (40%). I, Shown are immunoblot (IB) analyses of purified allergen‐expressing VNP (20 μg/lane), empty VNP (mock), or rArt v 1 probed with anti‐Art v 1 mAb and allergen‐expressing VNP (20 ?g/lane), empty VNP (mock) or buffer control probed with p30Gag mAb. kDa indicates molecular mass. J, Negative stain electron microscopy pictures of purified VNP. Data are representative (A, B, G‐J) or show the summary (C‐F) of three independent experiments (except one for G) performed in triplicates. Kruskal‐Wallis test followed by Dunn's correction. ns, not significant; ***P<0.001

Virus‐like nanoparticles are noninfectious viral core particles, surrounded by a lipid envelope derived from the host cell plasma membrane. We here differentially associated major pollen allergens of mugwort and birch, that is, human codon‐optimized(h)Art v 1 and hBet v 1, with VNP by fusing them N‐terminally to the MoMLV matrix protein (MA)p15^25^ or C‐terminally to the GPI anchor acceptor sequence of CD16b (Figure 1A) followed by expression analyses (Figure 1B and C)^37^ using the reagents listed in Table S1. Art v 1::GPI, in contrast to MA::Art v 1, revealed significant surface expression on 31.2 ± 11.9% of HEK‐293T transfectants (Figure 2A). Notably, MA::Art v 1 showed clear‐cut intracellular expression in the majority of transfectants upon permeabilization (99.6 ± 0.7) (Figure 2B). The anti‐Art v 1 mAb clone #5 recognizes an epitope on the Art v 1 molecule, which is not destroyed by the fixation and permeabilization procedure. Therefore, in these stainings also the Art v 1::GPI transfectants appear positive due to the simultaneous recognition of both surface and intracellular pools of the GPI‐anchored Art v 1 molecule. Control transfected HEK‐293T cells (empty pEAK12 plasmid, MA::Bet v 1 or Bet v 1::GPI) were not stained by the Art v 1 mAb (Figure 2C‐F), while both Bet v 1 fusion proteins (FP) were recognized by the Bet v 1‐specific mAb P6^39^ (not shown). Transfection with GFP was used as an independent positive control, which, however, lost its genuine fluorescence when cells were fixed for intracellular staining^40^ with the anti‐Art v 1 monoclonal antibody.

Membrane fractionation of HEK‐293T transfectants showed that both MA::Art v 1 (Figure 2G) and Art v 1::GPI (Figure 2H), similar to GPI‐anchored CD59 but in contrast to transmembrane CD147, are targeted to membrane lipid rafts (fractions #2 and #3) of gradients, which were confirmed in an independent assay^41^ (Figure S1A). Moreover, both Art v 1 FPs could be clearly detected in VNP preparations derived from MA::Art v 1‐ or Art v 1::GPI‐transfected HEK‐293T cells upon cotransfection with pMD.OGP (Figure 2I top panel). For SDS‐PAGE and subsequent immunoblotting, similar amounts of VNP (20 μg/lane) were resolved as proven by the presence of similar amounts of viral core protein Gag in the different preparations (Figure 2I bottom panel). Both allergen FPs had a higher molecular weight than recombinant Art v 1 due to their fusion to the GPI anchor or MA. Neither MA::Bet v 1 VNP nor Bet v 1::GPI VNP were recognized by the anti‐Art v 1 mAb, although similar amounts of these VNP were loaded onto SDS‐PAGE (Figure 2I bottom panel). Electron microscopy analyses revealed spherical structures of approximately 100 nm resembling VNP as described (Figure 2J).^24^ Dynamic light scattering determined a VNP size of 90.6 ± 22.7 nm for MA::Art v 1 VNP, 113.2 ± 16.1 nm for Art v 1::GPI VNP, and 131.3 ± 32.5 nm for empty VNP (Figure S1B), identifying VNP as “extrafine particles.”^42^ The surface zeta potential of VNP revealed −13.4 ± 1.1 mV for MA::Art v 1 VNP, −13.8 ± 1.6 mV for Art v 1::GPI VNP, and −13.0 ± 1.7 mV for empty VNP (Figure S1C) and was indicative for stable colloidal preparations.

### 3.2 In splenocyte preparations, VNP become preferentially engulfed by CD103^+^ DC and stimulate allergen‐specific CD4^+^ T cells

**Figure 3:**
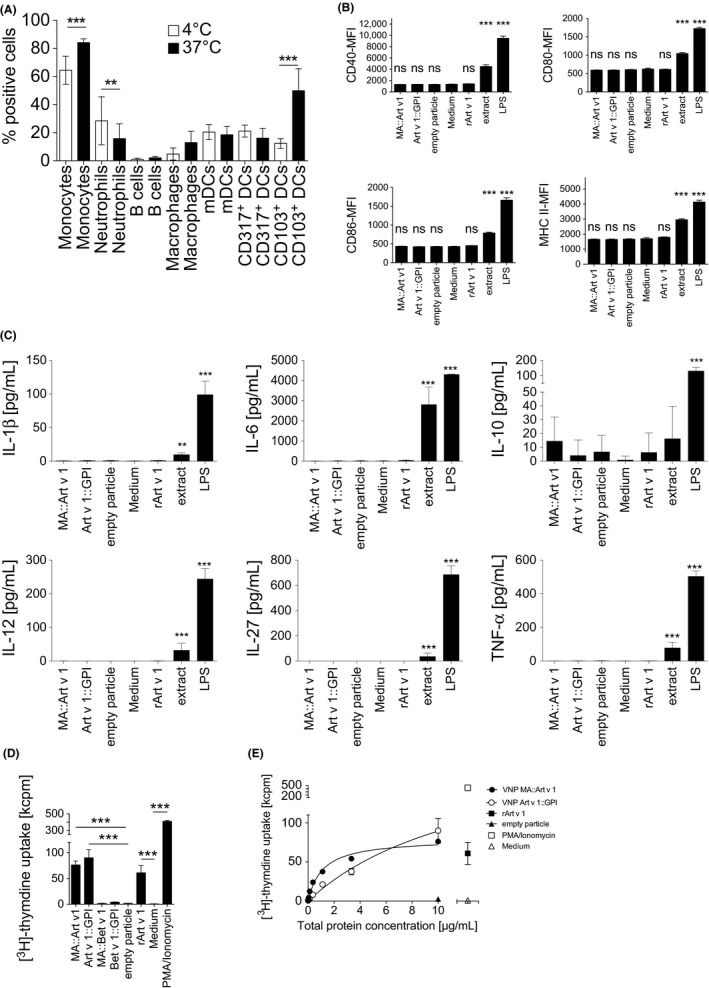
Uptake and immunostimulatory capacity of allergen‐expressing virus‐like nanoparticles (VNP). A, Binding and uptake of fluorescent MA::mCherry VNP by the indicated splenic cell types. Open bars indicate positive cells at 4°C, filled bars show positive cells at 37°C. B, Shown is flow cytometry expression of CD40, CD80, CD86, and MHC class II (HLA DR1) on bone marrow‐derived dendritic cells (BMDC) after co‐incubation with MA::Art v 1 VNP, Art v 1:: glycosylphosphatidylinositol (GPI) VNP, empty VNP (all at 10 μg/mL), medium alone, rArt v 1 (1 μg/mL), aqueous mugwort pollen extract (100 μg/mL), or LPS (100 ng/mL) for 24 h. C, Shown are the cytokine levels of IL‐1β, IL‐6, IL‐10, IL‐12, IL‐27, and TNF‐α secreted by BMDC (2 x 10^5^/well in 200 μL) upon co‐incubation with VNP expressing MA::Art v 1, Art v 1::GPI, empty particles, rArt v 1 (1 μg/mL), medium alone, aqueous mugwort extract (100 μg/mL), or LPS (100 ng/mL) for 24 h. D, Shown is proliferation of splenocytes of TCR/DR1 mice incubated with MA::Art v 1 VNP, Art v 1::GPI VNP, empty VNP (10 μg/mL), rArt v 1, (0.5 μg/mL), medium alone, or PMA/ionomycin (10^−7^M PMA, 120 ng/mL, respectively) for 72 h followed by a 16‐h methyl‐[3H]‐thymidine pulse (1 μCi/well). E, Shown is dose‐dependent proliferation of splenocytes from TCR/DR1 tg mice incubated with increasing amounts of MA::Art v 1 VNP or Art v 1::GPI VNP. Data show the summary of three independent experiments (A‐E) performed in triplicates. kcpm, kilo counts per minute; Kruskal‐Wallis test followed by Dunn's correction (A, D, E) or one‐way ANOVA followed by Tukey's correction (B, C). ns, not significant; **P<0.01; ***P<0.001

Next, we sought to clarify whether VNP expressing allergen FPs would activate allergen‐specific CD4^+^ T cells and whether this would be preceded by their binding and uptake and/or activation of antigen‐presenting cells (APC). Upon co‐incubation of splenocyte preparations with fluorescent MA::mCherry expressing VNP at 4°C, monocytes, followed by CD103^+^ myeloid dendritic cells (DC), bound VNP most efficiently. In contrast, CD103^+^ myeloid DC were most actively taking up VNP at 37°C when compared to other APC subsets (Figure 3A and Figure S3A). Notably, neither 24 (Figure 3B and Figure S2A) nor 48 (Figure S2B and C) hours of co‐incubation of MA::Art v 1 VNP, Art v 1::GPI VNP, empty VNP (10 μg/mL each), or rArt v 1 protein (1 μg/mL) with bone marrow‐derived dendritic cells (BMDC) of TCR/DR1 allergy mice upregulated CD40, CD80, CD86, or MHC class II expression, when compared to medium. However, BMDC co‐incubated with allergen‐expressing VNP secreted modest levels of IL‐10, while inflammatory cytokines (IL‐1β, IL‐6, IL‐12, IL‐27, or TNF‐α) were not modulated (Figure 3C and Figure S3B). Instead, LPS (100 ng/mL) and aqueous mugwort pollen extract (100 μg/mL) significantly upregulated all investigated activation markers and inflammatory cytokines (Figure 3B and C and Figure S3B).

To analyze their T cell stimulatory capabilities, we incubated splenocytes of Art v 1‐specific TCR/DR1 allergy mice with MA::Art v 1 VNP, Art v 1::GPI VNP, empty VNP (10 μg/mL), or rArt v 1 (0.5 μg/mL) and compared them to cultures supplemented with either medium alone or PMA plus ionomycin, respectively. Of note, both MA::Art v 1 VNP and Art v 1::GPI VNP, similar to rArt v 1, specifically stimulated T cell proliferation (Figure 3D). In contrast, neither empty VNP nor VNP expressing the control FPs MA::Bet v 1 or Bet v 1::GPI activated Art v 1‐specific T cells (Figure 3D). Dose‐response determinations revealed a better stimulatory capacity of MA::Art v 1 VNP (EC_50_ 1.1 ± 0.2 μg/mL) compared to Art v 1::GPI VNP (EC_50_ 13.6 ± 7.8 μg/mL) (Figure 3E and Table S2), most likely due to the higher concentration of Art v 1 in these VNP (Figure 2I), which is in line with results from allergen‐specific protein determinations performed with the Art v 1‐specific mAb clone #5 by ELISA. *In vitro*, both Art v 1‐expressing VNP, similar to rArt v 1, but in clear contrast to empty VNP or medium alone, stimulated the secretion of Th1, Th2, Th17, and regulatory cytokines (Figure S4). Notably, both MA::Art v 1 and Art v 1::GPI VNP promoted a Th1‐dominated immune response^43^ with Th1/Th2‐ratios of 27.8 ± 2.9 and 35.4 ± 3.6, respectively, for IFN‐γ/IL‐4 and 9.1 ± 0.9 and 11.3 ± 7.7, respectively, for IFN‐γ/IL‐13 (Figure S4B, E, H).

### 3.3 MA::Art v 1 VNP are hypoallergenic

**Figure 4:**
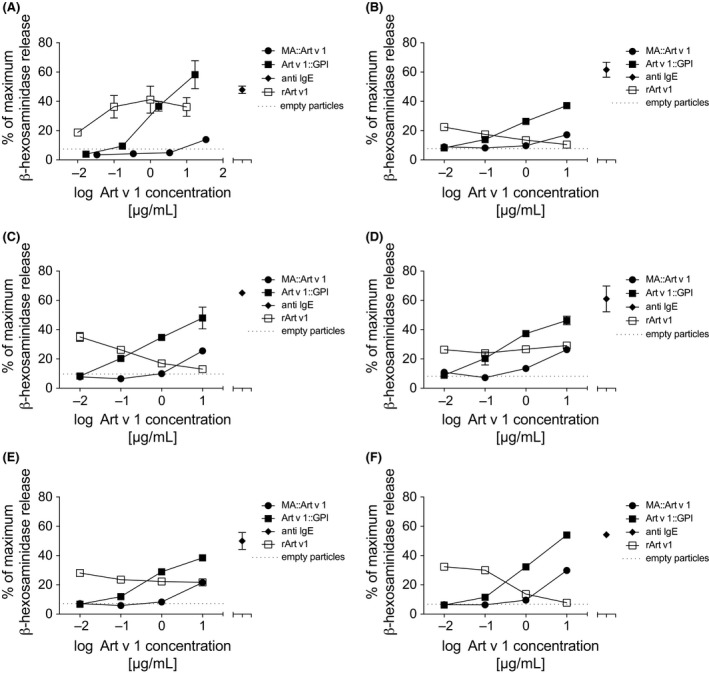
Shielded MA::Art v 1 expressing virus‐like nanoparticles (VNP) are hypoallergenic. A, Shown are β‐hexosaminidase release assays of rat basophil leukemia cells (RBL)‐2H3 cells sensitized with pooled mugwort‐specific mouse sera and incubated with the indicated concentrations of soluble rArt v 1 or VNP‐resident Art v 1, in the form of MA::Art v 1 or Art v 1:: glycosylphosphatidylinositol (GPI) VNP, respectively. Dotted line shows the maximum degree of β‐hexosaminidase release obtained upon incubation with 100 μg of empty VNP, which corresponds to the maximum protein concentrations of MA::Art v 1 or Art v 1::GPI VNP applied. B‐F; Shown are β‐hexosaminidase release assays of RS‐ATL8 cells sensitized with mugwort‐specific human sera from five allergic patients and incubated with the indicated concentrations of soluble rArt v 1 or VNP‐resident Art v 1, in the form of MA::Art v 1 or Art v 1::GPI VNP, respectively. Dotted line shows the maximum degree of β‐hexosaminidase release obtained upon incubation with 66 μg of empty VNP, which corresponds to the maximum protein concentrations of MA::Art v 1 or Art v 1::GPI VNP applied. Anti‐IgE mAbs were used at a fixed concentration of 10 μg/mL as FcεRI/IgE‐specific positive controls

To study the allergenic activity of Art v 1‐expressing VNP, we passively sensitized wild‐type rat basophil leukemia (RBL) cells or RS‐ATL8 cells expressing the human FcεRI^44^ with pooled, Art v 1‐specific mouse sera or sera of five mugwort allergic individuals, respectively, and incubated them with different doses of MA::Art v 1 VNP, Art v 1::GPI VNP, empty VNP, rArt v 1, or anti‐IgE. Figure 4A‐F and Table S3 show that MA::Art v 1 VNP, similar to empty VNP, were hardly able to stimulate β‐hexosaminidase release from sensitized RBL cells over the majority of concentrations tested. Only the highest MA::Art v 1 VNP concentrations tested induced modest levels of RBL degranulation. In contrast, Art v 1::GPI VNP, similar to rArt v 1 or anti‐IgE antibody, consistently activated RBL cells at three of four log_10_‐diluted concentrations tested. Quantitative ELISA revealed that the Art v 1 protein constitutes 17.8 ± 1.2% of the mass of Art v 1::GPI VNP (not shown). In RBL assays, Art v 1::GPI expressed on VNP was less active than soluble rArt v 1 suggesting hypoallergenicity of the Art v 1::GPI variant^45^, ^46^, ^47^ reminiscent of previously described hypoallergenic vaccine candidates either expressed on virus‐like particles, for example*,* for cat dander, or obtained upon trimerization of the immunodominant allergen, for example*,* for the major birch pollen allergen Bet v 1.^20^, ^48^

### 3.4 MA::Art v 1 VNP are nonimmunogenic and do not induce Art v 1‐specific antibodies *in vivo* and are taken up locally in the lung

**Figure 5:**
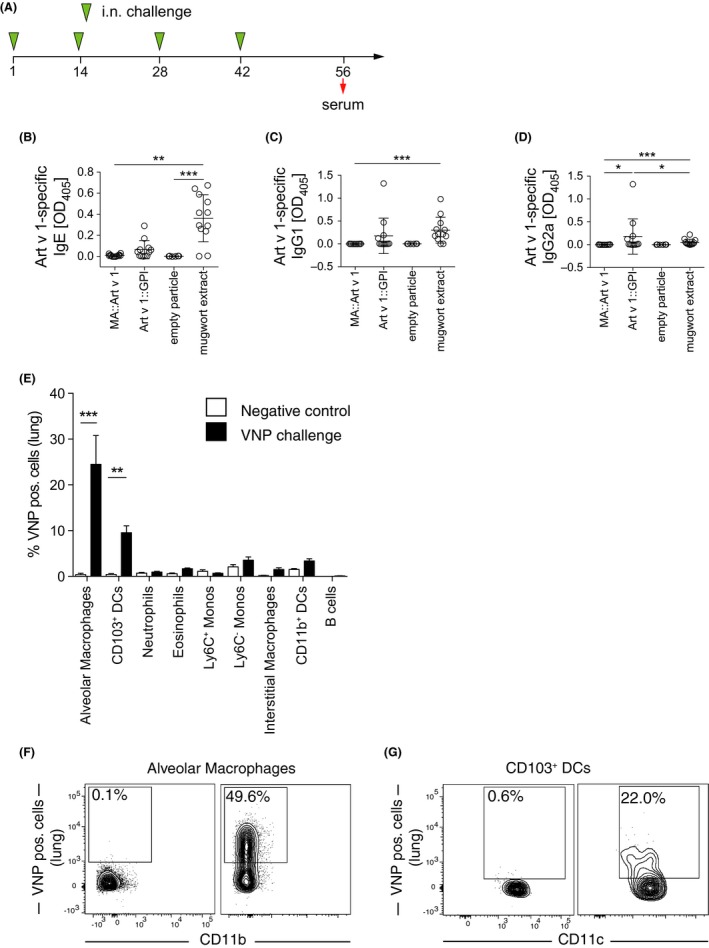
Significant induction of allergen‐specific immunoglobulins by virus‐like nanoparticles (VNP) expressing surface‐expressed but not shielded allergen. A, Scheme used to expose mice and to analyze sera. Shown are Art v 1‐specific serum levels (day 56) of B, IgE, C, IgG1, and D, IgG2a of TCR/DR1 mice, which were intranasally exposed to MA::Art v 1 or Art v 1:: glycosylphosphatidylinositol (GPI) expressing VNP, empty particles, or aqueous mugwort extract biweekly according to A. Each symbol represents an individual mouse. E, Shows the percentage of the indicated fluorescent cell populations in lung homogenates of mice treated with fluorescent VNP (black bars) or PBS (white bars). Representative FACS plots of either F, alveolar macrophages and G, CD103^+^ dendritic cells (DCs) in lung homogenates of mice treated with PBS (left panels) or fluorescent VNP (right panels). Data show the summary (B‐D) of 12 (except six for empty particles) treated mice per group of two independent (except one for empty particles) experiments. E shows the summary of one of two representative experiments (n = 5 mice per group). F and G show representative two‐parameter dot plots (n = 5 mice per group). Only significant differences are indicated in B to E. Kruskal‐Wallis test followed by Dunn's multiple comparison. *P<0.05; **P<0.01; ***P<0.001

To determine the sensitizing potential of allergen‐expressing VNP, we i.n. exposed TCR/DR1 allergy mice to MA::Art v 1 VNP, Art v 1::GPI VNP, empty VNP, or aqueous mugwort pollen extract four times in biweekly intervals (Figure 5A). I.n. administration was chosen because it directly targets the specific organs of mugwort allergy (ie, nose, airways, and lung), and nasal sprays have proven successful, for example, for the prevention of airborne infectious diseases (FluMist, influenza^49^). MA::Art v 1 VNP, as opposed to aqueous mugwort pollen extract, neither induced Art v 1‐specific IgE nor IgG (Figure 5B‐D). In contrast, some of the mice exposed to Art v 1::GPI VNP, but none of those exposed to empty VNP, developed modest, allergen‐specific IgE, IgG1, and IgG2a levels (Figure 5B‐D). The fact that Art v 1::GPI VNP have the principle capacity to sensitize some of the tested mice questioned their suitability as prophylactic vaccine already at this stage. While empty VNP were recognized by the MoMLV p30Gag‐specific mAb R187 in immunoblots (Figure S5A) and ELISA (Figure S5B), no such reactivity was detectable in mouse sera (Figure S5C‐E). *In vivo* uptake in lungs of fluorescently labeled VNP was dominated by alveolar macrophages and CD103^+^ DCs (Figure 5E‐G). Preliminary data suggest that CD103^+^ DC are less potent in taking up free allergen (not shown). Apart from the i.n., also the s.c. route was evaluated for prophylactic VNP application. However, under these conditions, even MA::Art v 1 VNP turned out to be sensitizing as they induced allergen‐specific IgE already after three s.c. injections. IgE levels further increased upon subsequent i.n. and intratracheal challenges with the allergen extract (not shown).

### 3.5 Preventive treatment with MA::Art v 1 expressing VNP protects from subsequent allergic sensitization and ameliorates lung function

**Figure 6:**
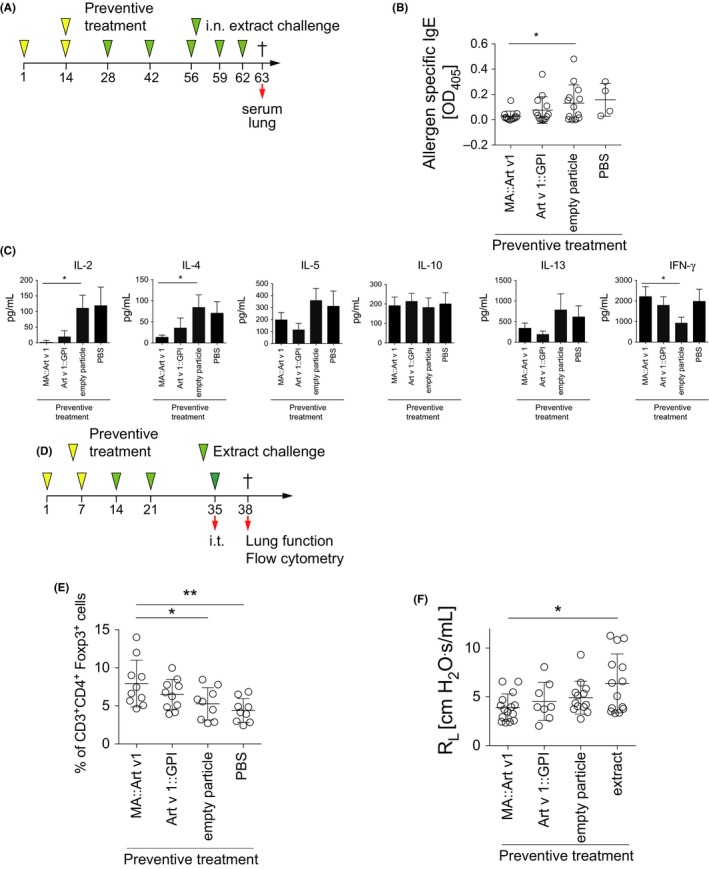
Preventive treatment of mice with allergen‐specific virus‐like nanoparticles (VNP). A, Scheme for preventive treatment and challenge of mice in B‐C. B, Art v 1‐specific serum IgE levels (day 63) of TCR/DR1 mice i.n. prophylactically treated with MA::Art v 1 VNP, Art v 1:: glycosylphosphatidylinositol (GPI) VNP, empty VNP, or PBS and challenged as indicated. C, Cytokines produced by lung homogenates of mice (day 63) upon restimulation with aqueous mugwort pollen extract (100 μg/mL for 72 h). D, Scheme of respiratory exposure and challenge protocol. E, Relative numbers of CD3^+^ CD4^+^Foxp3^+^ cells in lung homogenates and F, lung resistance of mice prophylactically treated and challenged as in D. Data show the summary (B‐C) of 13 (except four for PBS) mice per group of three (except one for PBS) independent experiments or the summary (E) of 10 (except nine for empty VNP and PBS) mice of two independent experiments. F shows the summary of 15 (except 14 for PBS, 13 for empty particles, and 8 for Art v 1::GPI) mice of three independent experiments (except two for Art v 1::GPI). Only significant differences are indicated. One‐way ANOVA followed by Holm's Sidak correction. *P<0.05; **P<0.01

As MA::Art v 1 VNP were neither immunogenic nor allergenic but clearly activated allergen‐specific CD4^+^ T cells *in vitro* (Figure 3D‐E), we hypothesized that the *in vivo* application of MA::Art v 1 VNP might inhibit subsequent sensitization with mugwort pollen extract, akin to the mechanisms observed in peptide immunotherapy of allergy.^50^ To test this, we i.n. exposed mice to MA::Art v 1 VNP, Art v 1::GPI VNP, empty VNP or PBS twice, separated by a biweekly interval followed by five challenges with aqueous mugwort pollen extract (Figure 6A), known to induce fairly robust allergen‐specific IgE titers and also allowing to monitor cytokine recall responses by lung cells. We found that, in principle, mice exposed to MA::Art v 1 VNP during preventive treatment did not produce allergen‐specific IgE in response to allergen challenge (only 1 of 15 mice showed a low specific IgE titer) when compared to mice exposed to empty VNP (7 of 13 mice produced IgE, *P* = 0.01) or PBS, respectively (Figure 6B). In contrast, preventive treatment with Art v 1::GPI VNP could not completely protect from the induction of specific IgE in all animals (4 of 13 mice with allergen‐specific IgE) (Figure 6B and Figure S6A and B).

Notably, restimulation of lung homogenates derived from the four groups of mice with aqueous mugwort extract revealed a clear decrease in Th2‐associated cytokines in mice which had been prophylactically treated with MA::Art v 1 VNP compared to those exposed to empty VNP (Figure 6C). In contrast, levels of IFN‐γ and IL‐10 were found to be equal or even significantly elevated by preventive treatment with MA::Art v 1 VNP, indicative of a Th1/Treg‐prone immune response (Figure 6C). Comparable data were obtained when lung T cells were restimulated with the immunodominant Art v 1_23‐35_ peptide (Figure S6C). Spurred by these observations and cognizant that Tregs are key modulators of allergen‐specific immune responses,^30^, ^31^, ^32^, ^51^ we considered that preventive treatment with MA::Art v 1 VNP may increase the proportion of lung‐resident CD3^+^CD4^+^Foxp3^+^ Tregs. Due to the different endpoints measured in these experiments, increased sensitivity could be achieved by shorter intervals between allergen exposures and a final intratracheal booster with allergen.^52^ Investigation of lung homogenates of mice prophylactically treated with VNP and challenged with aqueous mugwort pollen extract as indicated (Figure 6D) showed significantly increased CD3^+^CD4^+^Foxp3^+^ Treg numbers in mice prophylactically treated with MA::Art v 1 VNP as compared to mice treated with empty VNP (Figure 6E and Figure S7A and B). Consistently, methacholine‐induced lung resistance was lowest in mice prophylactically treated with MA::Art v 1 VNP (Figure 6F).

## 4 DISCUSSION

We report the development of a new platform for the prophylactic induction of allergen‐specific tolerance which is based on shielding of immunodominant, full‐length allergens inside of VNP. Such allergen‐expressing VNP are (a) hardly able to degranulate RBL cells sensitized with allergen‐specific human or mouse IgE (ie, they are hypoallergenic), (b) they do not induce allergen‐specific IgE or IgG *in vivo* (ie, they are non‐immunogenic), however, they are (c) regularly being taken up and processed by professional APCs, and (d) activate allergen‐specific CD4^+^ T cells *in vitro*. Importantly, we showed that MA::Art v 1 VNP, when prophylactically (i.n.) applied, (e) increase the fraction of CD3^+^CD4^+^Foxp3^+^ Tregs in lungs of allergen‐challenged mice. Moreover, (f) the increased Treg numbers correlate with the repression of organ(lung)‐specific Th2 cytokine production and (g) AHR upon challenge. As allergy prevention in high‐risk populations via exposure of the target organ to the culprit allergens is now emerging as a novel, attractive and ethically responsible approach,^16^, ^17^, ^53^ therapeutic administration of the here developed VNP was not part of this study.

We here confirm that the VNP‐delivered allergens are critically important for the immunoregulatory properties of VNP, as only the preventive treatment with allergen‐expressing, but not empty VNP or PBS, led to downregulation of IL‐2, IL‐4, IL‐5, and IL‐13 secretion by lung‐resident T cells and increases in Foxp3^+^ Tregs upon rechallenge *in vivo*. To the best of our knowledge, no allergen‐expressing particles have been developed so far, which induce suppression of allergen‐specific immune responses in the absence of inducing antibody responses. By contrast, it has been shown that they are highly immunogenic and induce strong allergen‐specific IgG responses while being deficient in inducing allergen‐specific T cell tolerance.^20^, ^21^ Whether a tolerogenic or immunogenic effect is achieved may therefore depend on the type of VNP used to deliver allergens and/or on the mode of administration. It is tempting to speculate that the T cell‐instructing APC, which have engulfed the allergen‐expressing VNP, might trigger specific T cells at suboptimal/differential conditions known to shut‐off IL‐2 and Th2 cytokine production while favoring a Tr1‐like cytokine environment rich in IFN‐γ and IL‐10.^29^, ^54^ Alternatively, the almost complete lack of IL‐2 might also result from consumption by the elevated numbers of Treg cells.^55^

How can the Treg‐inducing capability of the here created VNP be explained? Our observation that *in vivo* alveolar macrophages as well as CD103^+^ DC, compared to other APCs, significantly took up VNP (Figures 3A and 5E) offers a plausible explanation. Both alveolar macrophages, defined as CD11b^−^CD11c^+^SiglecF^+^ cells,^56^, ^57^ and CD11c^+^CD103^+^ DCs, are potent inducers of Foxp3^+^ Tregs in the lung^58^, ^59^ due to their capability to express retinaldehyde dehydrogenase 2, a key enzyme for the production of retinoic acid (RA) which synergizes with TGF‐β (and IL‐10) to induce Foxp3^+^ Treg.^57^, ^58^, ^60^ RA can also be induced by inhaled antigens^59^ and contributes to the homeostatic, anti‐inflammatory role of lung‐resident CD103^+^ DCs.^61^ Moreover, VNP are not regarded as “dangerous” by inflammatory‐type DCs, as no evidence for activation (Figure 3B and Figure S2A‐C) or secretion of inflammatory cytokines (Figure 3C and Figure S3B) was found upon coculture of allergen‐expressing or empty VNP with BMDC. In marked contrast, LPS regularly upregulated all four activation markers on DC and induced the secretion of a whole collection of inflammatory cytokines. This clearly indicated that VNP preparations are free of LPS and that the VNP backbone itself does not express activating DC ligands. The latter is not entirely surprising as HEK293 cells are used as producer cells, for example, for the production of γ‐retroviruses applied for (experimental) gene transfer in humans.^62^, ^63^

Our MoMLV‐based VNP are clearly different from the Qβ bacteriophage‐based VNP described earlier.^64^, ^65^ While Qβ‐based VNP express foreign antigens/allergens exclusively as surface‐exposed proteins/peptides on nonenveloped particles, the here described VNP can accommodate both surface‐exposed and shielded allergens. We here fused full‐length major allergens to the MoMLV MA p15, which generates a post‐translationally lipid‐modified MA::allergen‐fusion protein, robustly targeted to the inner leaflet of the lipid bilayer envelope of budding VNP.^25^ This strategy yields VNP‐containing major allergens, which are efficiently shielded from the environment and prevents not only their induction of allergen‐specific immunoglobulin responses but also their recognition by preformed IgE bound to FcεRI and thus basophil degranulation. This is in contrast to Art v 1::GPI VNP and Qβ particles, which express surface‐exposed allergen accessible (although at reduced levels) by FcεRI‐bound IgE (Schmitz et al.^20^ and Figure 4A‐F) bearing the potential risk of inducing anaphylaxis when applied to sensitized individuals *in vivo*. Although Art v 1::GPI VNP clearly activate basophil degranulation, their allergenic activity seems to be reduced when compared to equimolar amounts of soluble rArt v 1 (Figure 4A‐F). The restricted accessibility of effector cell‐bound IgE to recombinant allergens as well as the formation of large aggregates might influence their allergenicity as shown, for example, for Bet v 1 trimers previously.^48^ Significant contribution of cleaved, soluble forms of Art v 1::GPI molecules to the RBL degranulating activity of Art v 1:GPI VNP preparations was excluded by experiments in which either freshly prepared and washed VNP were used for assays or the buffer solution used for resuspending and storing of Art v 1::GPI VNP was entirely removed and exchanged by size exclusion centrifugation, ultracentrifugation, or both, directly before performing the RBL assays. Such experiments did not reveal differences in RBL degranulation induced by the differently prepared/purified VNP, indicating that Art v 1::GPI associated with the VNP surface is stable and the only relevant stimulating principle. Apart from that, the here described VNP also seem to be poor B cell antigens as neither prophylactic treatment with allergen‐expressing nor empty VNP led to significant antibody responses against VNP constituents themselves as shown by Western blotting (Figure S5A) and ELISA (Figure S5B‐E). In addition, no antibody responses directed against the MA‐ or GPI‐fusion partners used to attach Art v 1 to the inner or outer side of VNP could be observed (not shown). Nevertheless, as Art v 1::GPI VNP had the principle capacity to sensitize at least some of the prophylactically treated mice, they are considered inappropriate candidates for a prophylactic vaccine formulation.

Why empty VNP ameliorated lung resistance to a certain degree remains to be elucidated in future experiments. One possibility entertained in the past for the beneficial impacts on allergic symptoms of CpG‐rich VNP lacking allergen was their engagement of pattern recognition receptors on both innate and adaptive immune cells.^66^ So far, we did not find evidence that VNP (empty or allergen‐specific) would activate DC (Figure 3B and Figure S2A‐C); however, future studies will address that question in greater detail.

What could be the advantage of applying allergen‐expressing VNP over artificial liposomes containing recombinant allergens for vaccination purposes? VNP copackage complement regulatory proteins, for example, CD55 and CD59 “borrowed” from HEK‐293T cells (Figure 2G and H), possibly preventing immediate complement attack and thus premature degradation upon *in vivo* application. Moreover, VNP can easily accommodate other lipid‐modified, immunoregulatory molecules of choice, for example*,* cytokines and growth factors impacting on DC activation and differentiation or shaping T_eff_ cell programs. 29, 67 Moreover, oligo‐ or poly‐valent VNP could be generated, for example*,* by coexpression of several MA::allergen FPs or by concatenation of two or more allergens with MA. During VNP generation, the processes of allergen expression and envelopment are separately regulated, making the introduced allergen sequences amenable to facile manipulation (eg, display hypoallergens or altered peptide ligands^68^) and allowing for the introduction of further bioactive molecules (eg, regulatory proteins and/or (ribo)nucleic acids^69^).

In summary, allergen‐specific VNP expressing shielded cargo are novel formulations with high potential for allergen‐specific prevention of IgE sensitization and may be used as prophylactic allergy vaccines.

## CONFLICTS OF INTEREST

With regard to the authors’ disclosure of potential conflict of interests, we would like to indicate that Winfried F. Pickl holds stocks of Biomay AG and receives honoraria from Novartis and Rudolf Valenta has received research grants from Biomay AG and Viravaxx, Vienna, Austria, and serves as a consultant for Biomay AG, Viravaxx and Boehringer Ingelheim, Biberach, Germany. All other authors have no additional financial interests.

## AUTHOR CONTRIBUTIONS

B. K., R. V., and W. F. P. designed research; B. K., C. K., S. H., U. S., D. T., J. I., D. P., P. K., P. G., I. M., B. L., and A. N. performed research and analyzed data; R. V., A. E‐B., A.N., and W. F. P. supervised experiments; B. J‐S and G. G. provided critical reagents; B. K., U. S., R. V., and W. F. P. wrote the manuscript.
