# Supplementary figures and images for: Prevention of allergy by virus‐like nanoparticles (VNP) delivering shielded versions of major allergens in a humanized murine allergy model
Source: Allergy. 2018 Nov 5;74(2):246–60. doi: 10.1111/all.13573 (PMC6587790; doi:10.1111/all.13573)

Figure S1

A

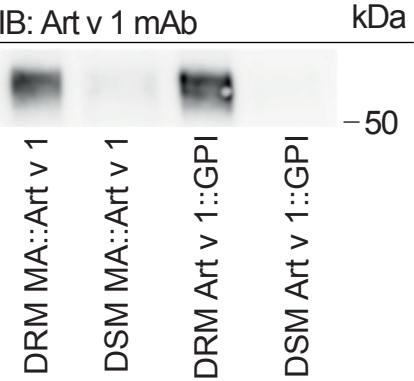

B

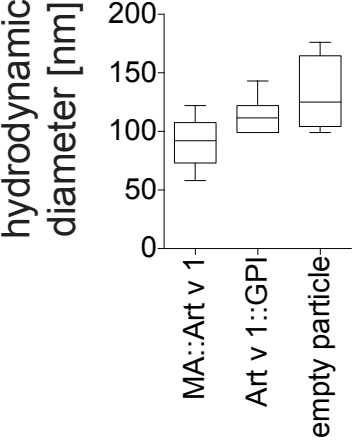

C

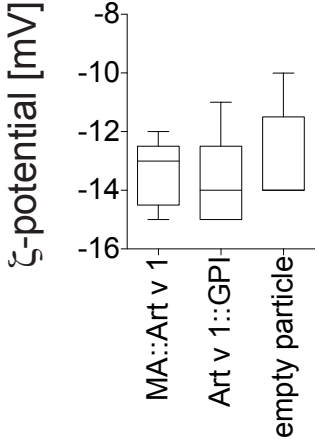

Supplement: Supplementary file 1 [file ALL-74-246-s001.pdf]

Figure S2

A

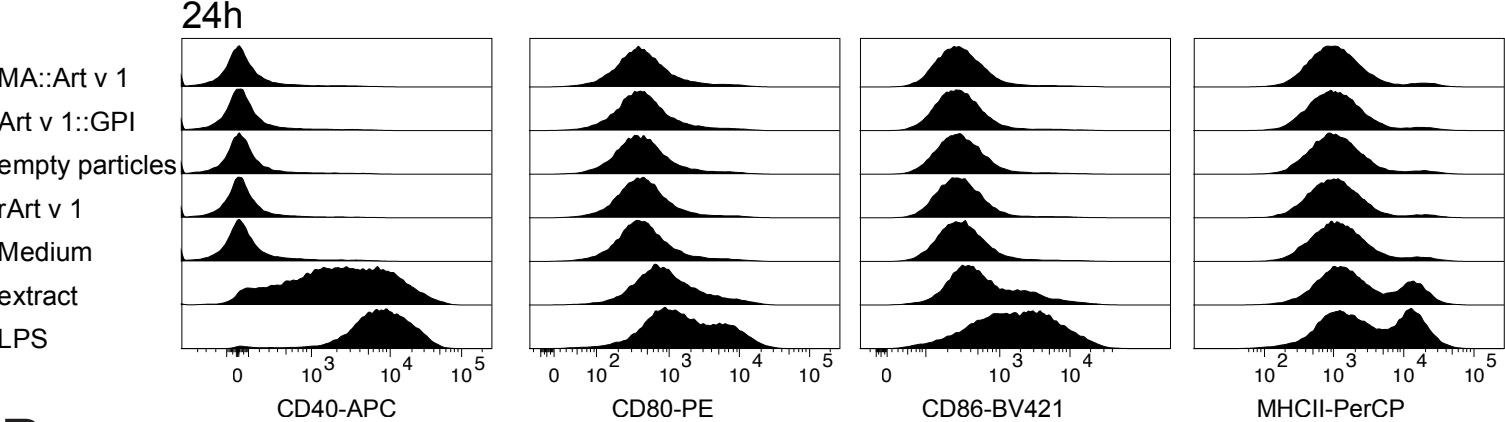

B

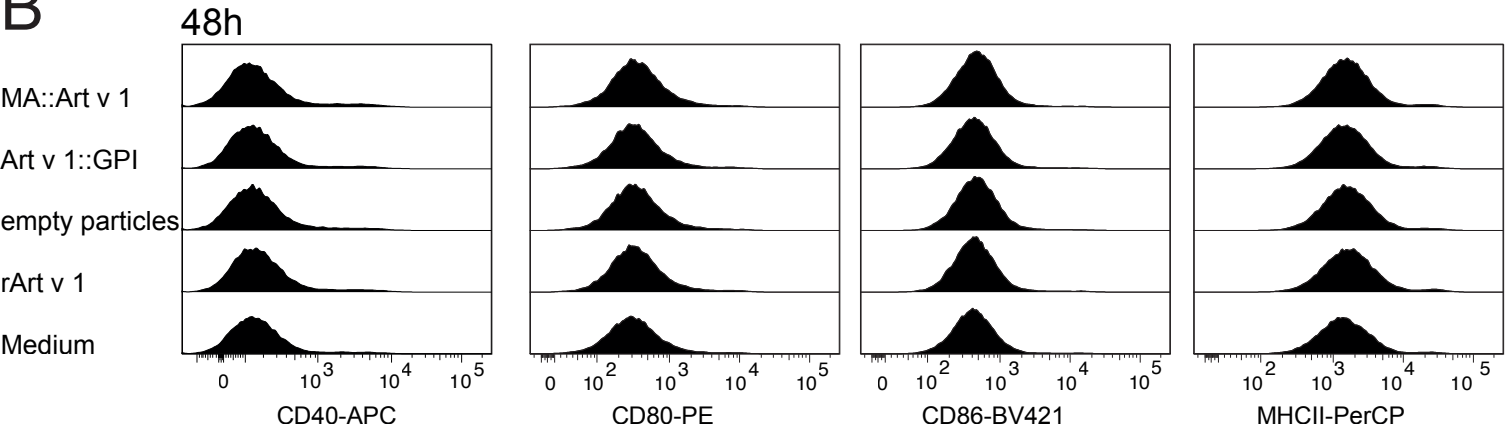

C

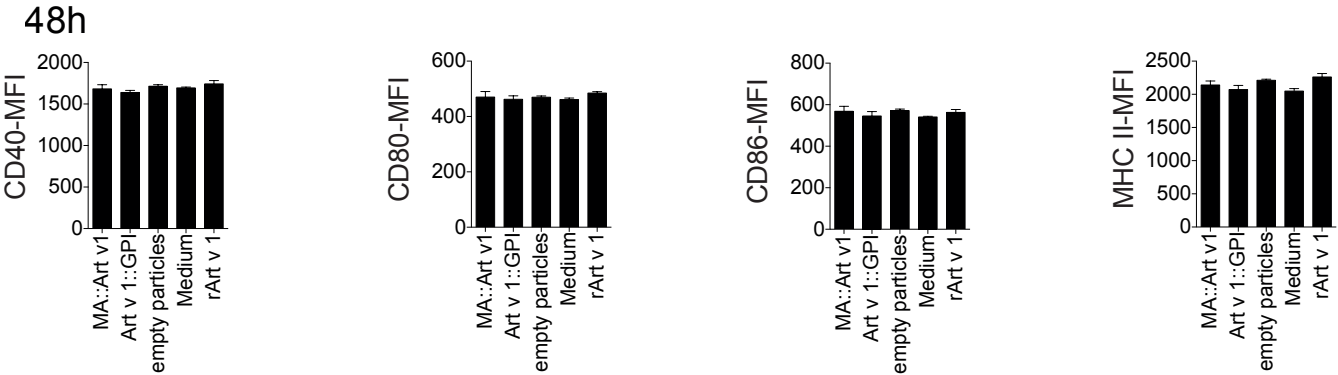

Supplement: Supplementary file 2 [file ALL-74-246-s002.pdf]

Figure S3

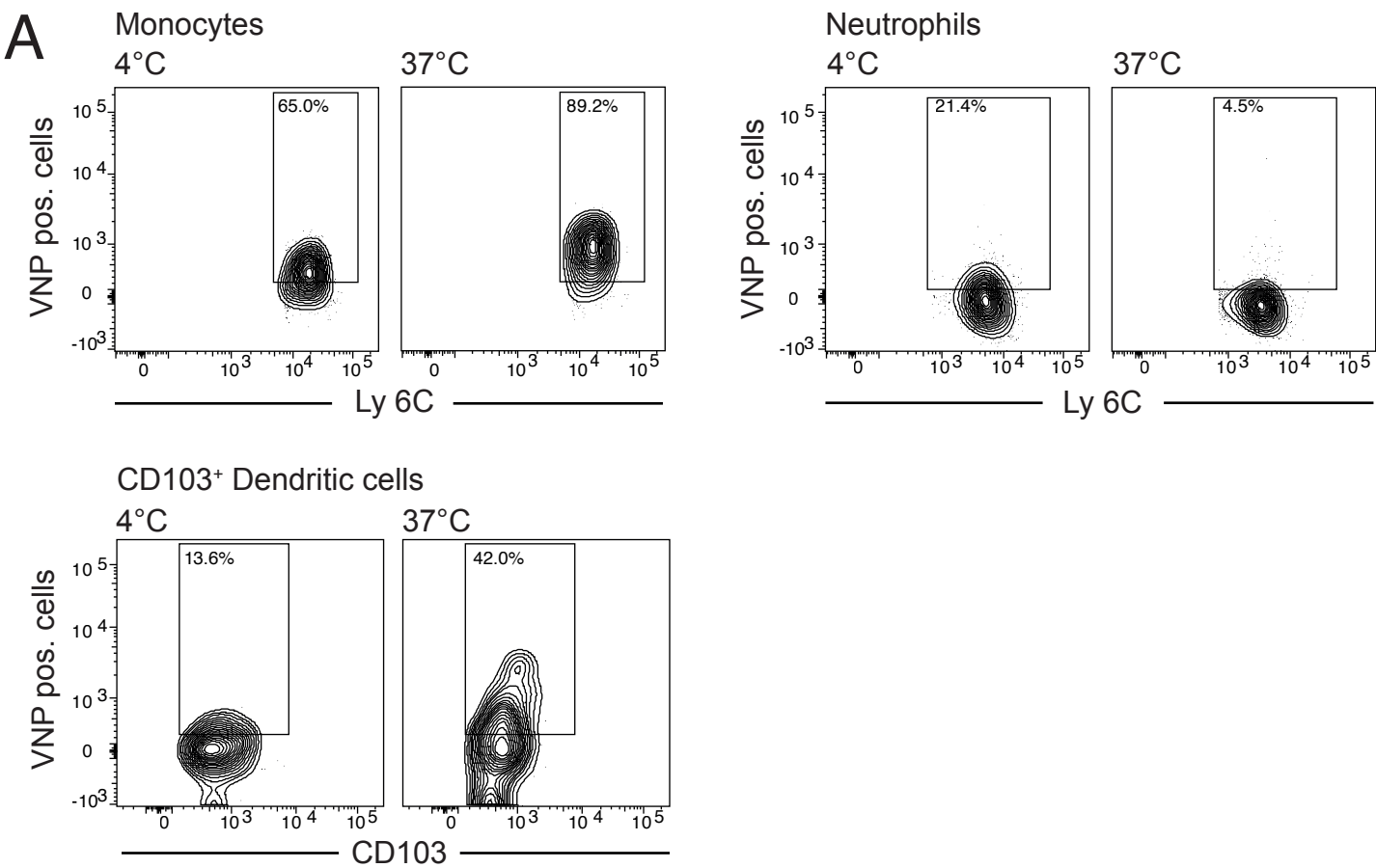

**B**

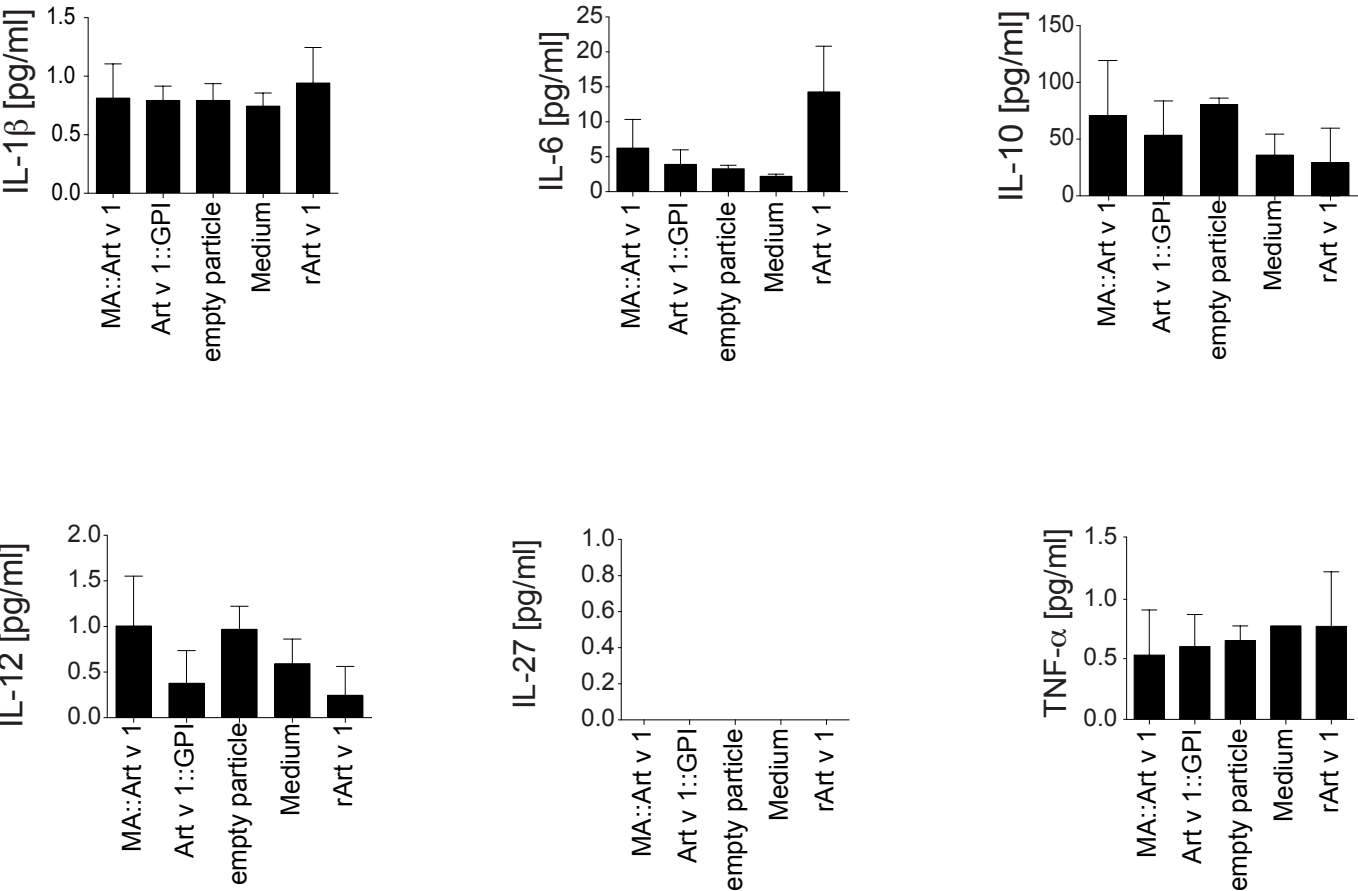

Supplement: Supplementary file 3 [file ALL-74-246-s003.pdf]

Figure S4

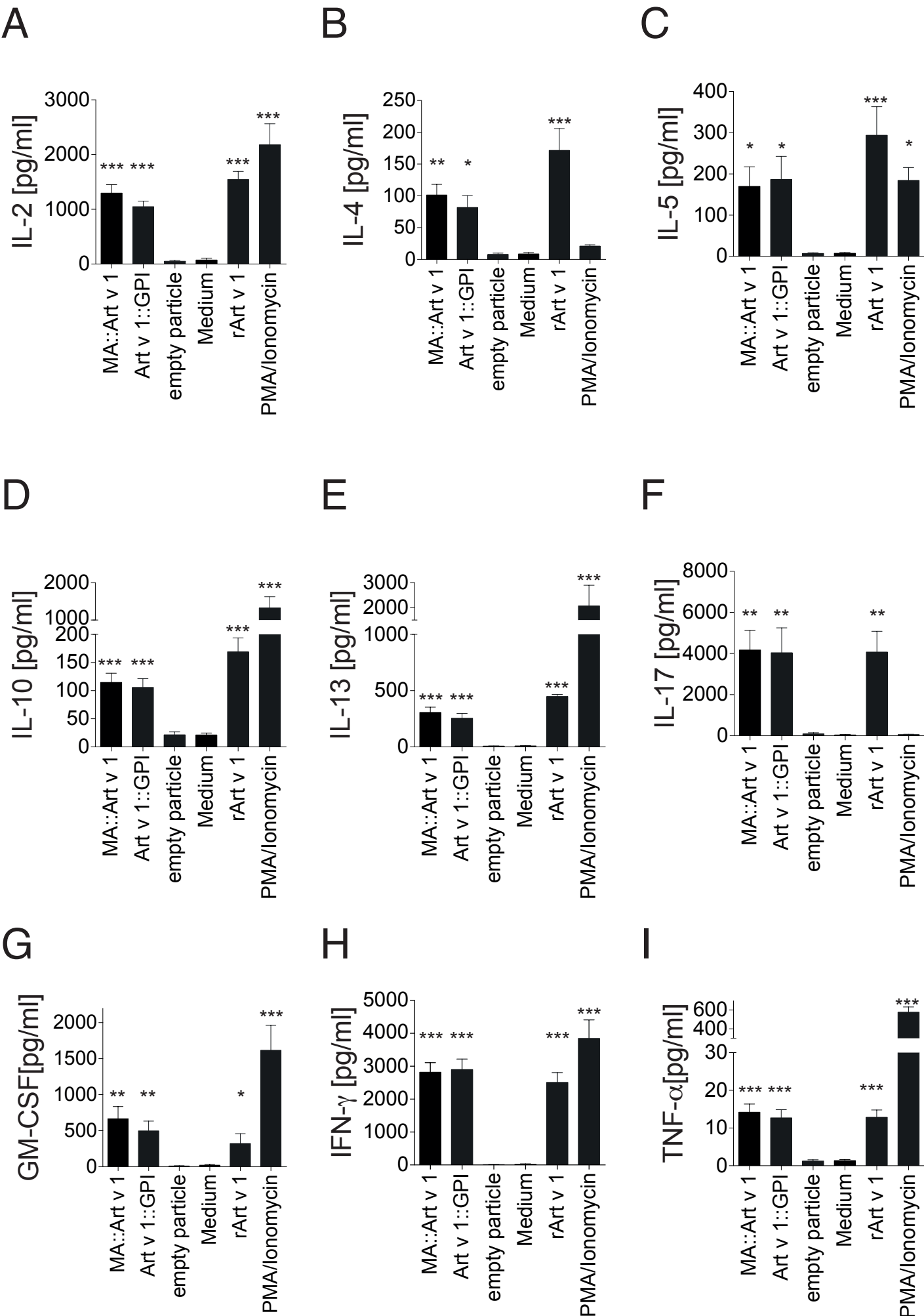

Supplement: Supplementary file 4 [file ALL-74-246-s004.pdf]

Figure S5

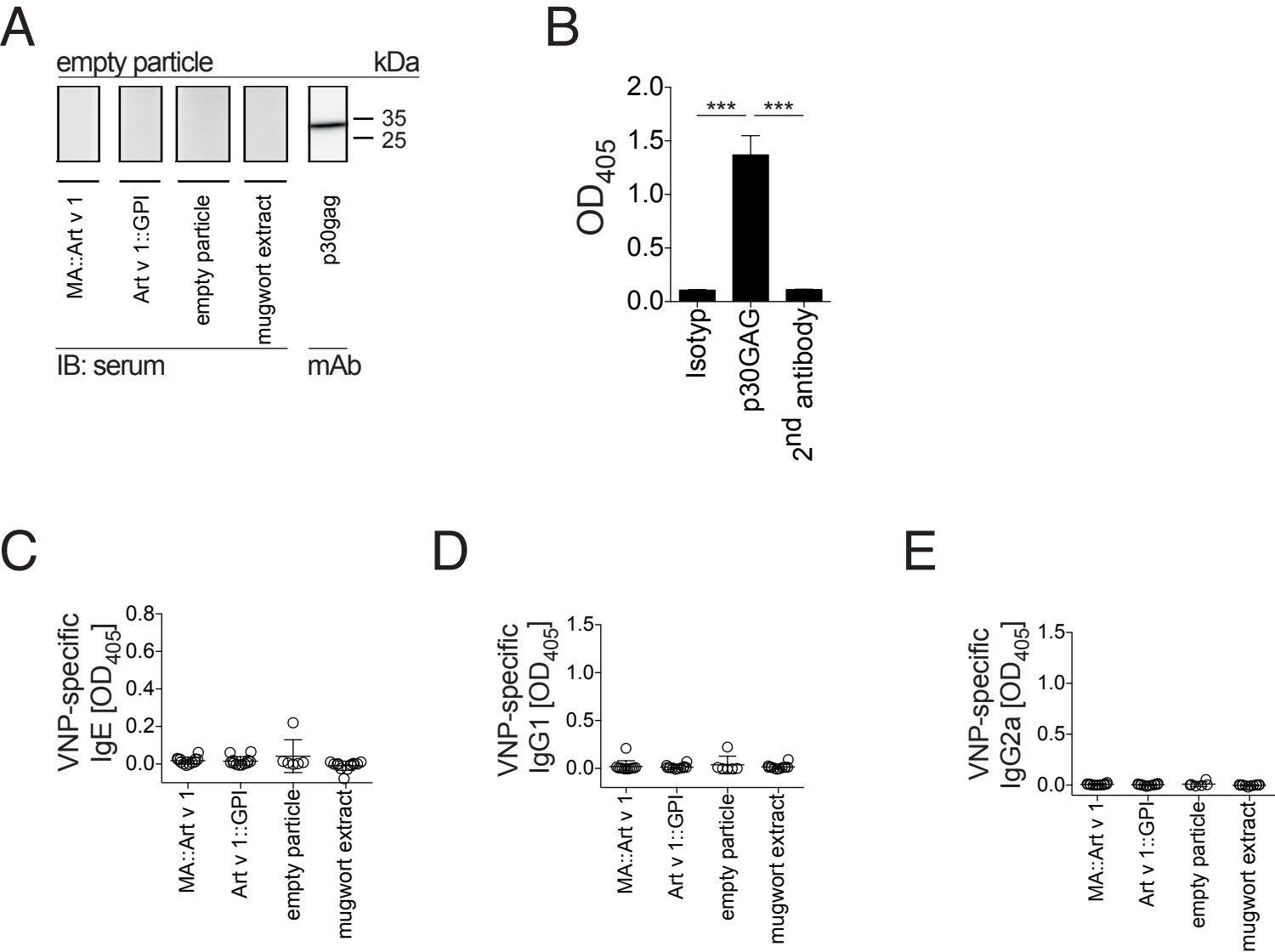

Supplement: Supplementary file 5 [file ALL-74-246-s005.pdf]

Figure S6

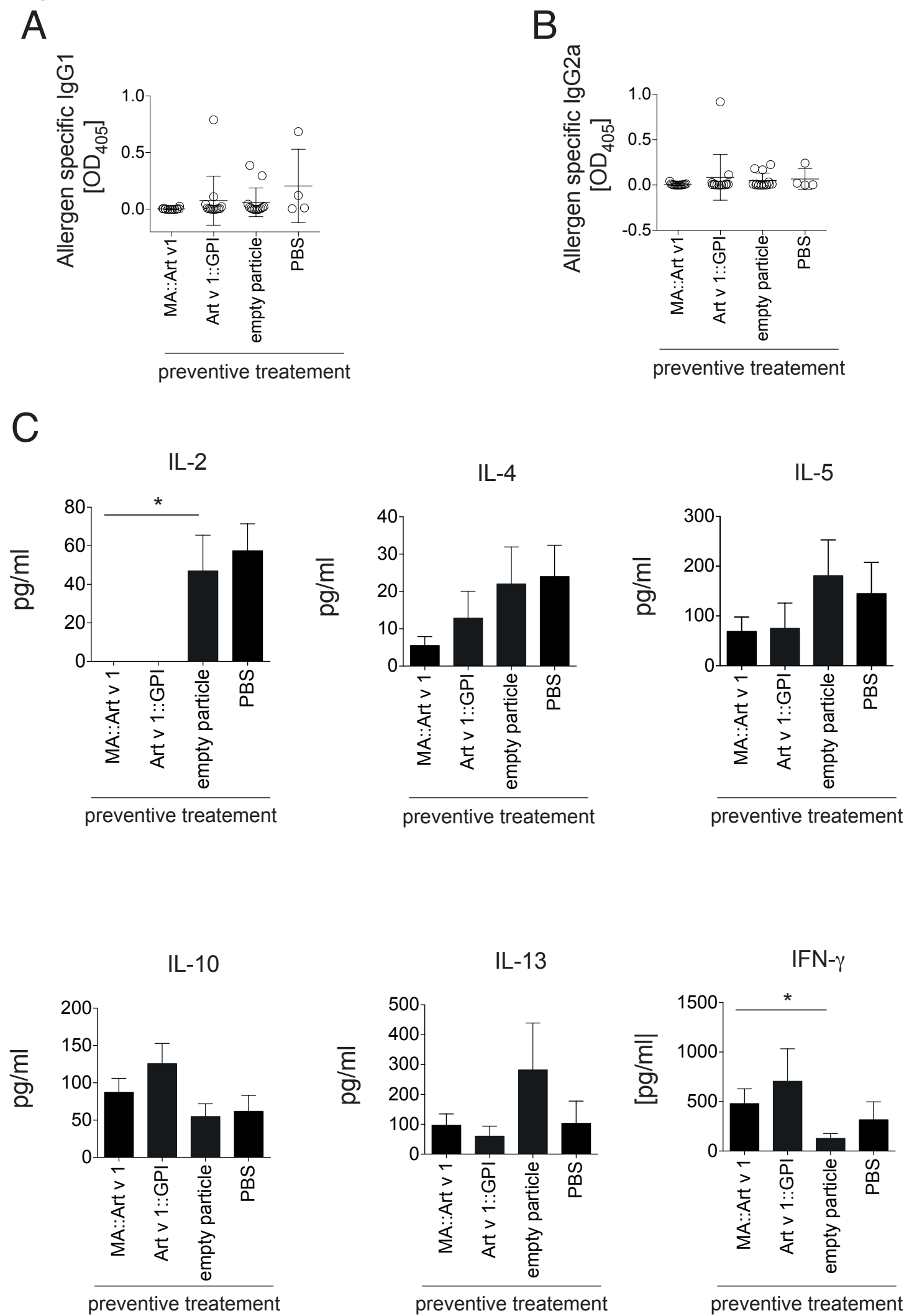

Supplement: Supplementary file 6 [file ALL-74-246-s006.pdf]

Figure S7

A

Gating strategy:

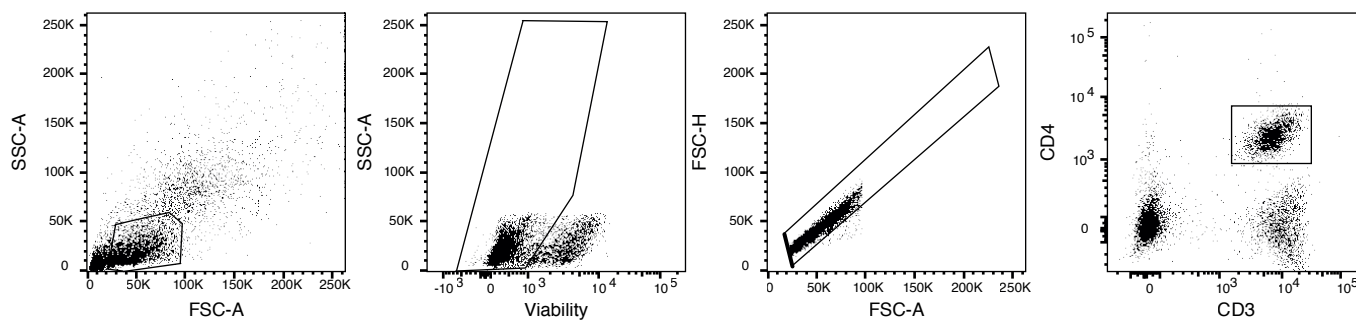

B

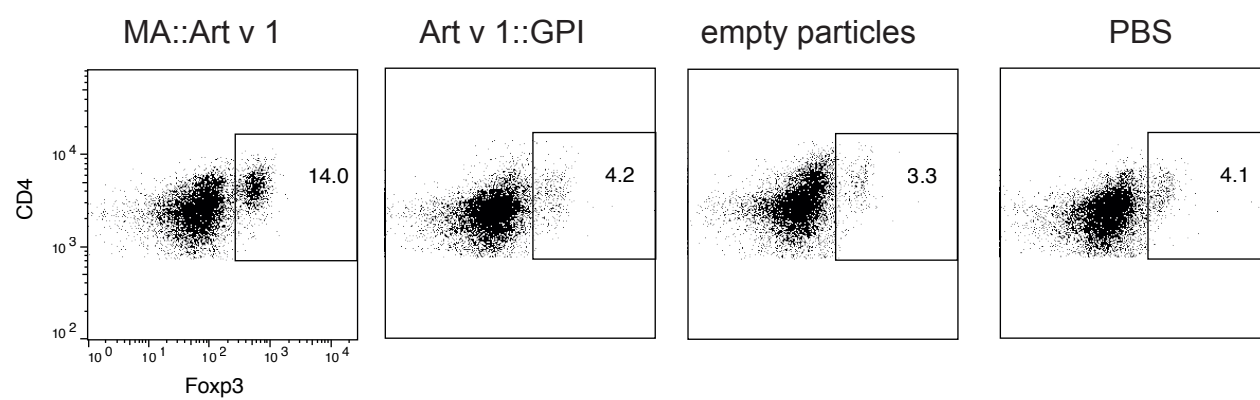

Supplement: Supplementary file 7 [file ALL-74-246-s007.pdf]
