# Supplementary material for: Prevention of allergy by virus‐like nanoparticles (VNP) delivering shielded versions of major allergens in a humanized murine allergy model
Source: Allergy. 2018 Nov 5;74(2):246–60. doi: 10.1111/all.13573 (PMC6587790; doi:10.1111/all.13573)
Supplement: Supplementary file 8 [file ALL-74-246-s008.docx]

**Table S1. List of antibodies used in this study**

| **Specificity** | **Clone name** | **Species** | **conjugated to** | **Source** | |  |
| --- | --- | --- | --- | --- | --- | --- |
| *Flow cytometry of producer cell lines* | | | | | | |
| Art v 1 | Clone 5 | mouse | - | R. Weiss, Salzburg, AUT | |  |
| Bet v 1 | P6 | mouse | - | R. Valenta, Vienna, AUT | |  |
| Mouse IgM | polyclonal | goat | AF 647 | Thermo Scientific, Waltham, MA, USA | |  |
| control | BIH0108 | mouse | - | One lambda, Waltham, MA, USA | |  |
| *Immunoblotting* |  |  |  |  |  |  |
| Art v 1 | Clone 5 | mouse | - | R. Weiss, Salzburg, AUT |  |  |
| CD59 | MEM-43/5 | mouse | - | Exbio, Praha, Czech Republic |  |  |
| CD147 | MEM-6/2 | mouse | - | Exbio, Praha, Czech Republic |  |  |
| Mouse Ig | polyclonal | goat | HRP | Dako, Santa Clara, CA, USA |  |  |
| *Art v 1-specific ELISA* | | | | | |  |
| Mouse IgE | R35-72 | rat | - | Becton Dickinson, Palo Alto, CA | |  |
| Mouse IgG2a | R19-15 | rat | - | Becton Dickinson, Palo Alto, CA | |  |
| Mouse IgG1 | A85-1 | rat | - | Becton Dickinson, Palo Alto, CA | |  |
| Rat Ig | polyclonal | goat | HRP | Thermo Scientific, Waltham, MA, USA | |  |
| *RBL assays* |  |  |  |  | |  |
| Mouse IgE | R35-72 | rat | - | Becton Dickinson, Palo Alto, CA | |  |
| Human IgE | E124.2.8 | mouse | - | Beckman Coulter, Brea, CA | |  |
| *Flow cytometric analysis of BM-DCs* | | | | |  |  |
| CD11c | HL3 | hamster | APC-Cy7- | Becton Dickinson, Palo Alto, CA |  |  |
| CD11b | M1/70 | rat | Alexa Fluor 700 | eBioscience, San Diego, CA, USA |  |  |
| CD40 | HM40-3 | hamster | APC | eBioscience, San Diego, CA, USA |  |  |
| CD80 | 16-10A1 | hamster | PE | eBioscience, San Diego, CA, USA |  |  |
| CD86 | GL-1 | rat | eFluor 450 | eBioscience, San Diego, CA, USA |  |  |
| HLA-DR | L243 | mouse | PerCP | BioLegend, San Diego, CA, USA |  |  |
| CD16/32 | 2.4G2 | rat | - | Becton Dickinson, Palo, Alto, CA |  |  |
| *Flow cytometric analysis of Splenocytes and lung homogenates* | | | | |  |  |
| CD11c | HL3 | hamster | APC-Cy7- | Becton Dickinson, Palo Alto, CA |  |  |
| CD11b | M1/70 | rat | PE-Cy7 | BioLegend, San Diego, CA, USA |  |  |
| LY6C | HK1.4 | rat | BV421 | BioLegend, San Diego, CA, USA |  |  |
| LY6G | 1A8 | rat | BV605 | BioLegend, San Diego, CA, USA |  |  |
| MHC II | M5/114.15.2 | rat | BV650 | BioLegend, San Diego, CA, USA |  |  |
| CD103 | 2E7 | hamster | PerCP-Cy5.5 | BioLegend, San Diego, CA, USA |  |  |
| CD317 | 927 | rat | APC | BioLegend, San Diego, CA, USA |  |  |
| CD19 | 1D3 | rat | PE | BioLegend, San Diego, CA, USA |  |  |
| SiglecF | E50-2440 | rat | AF647 | Becton Dickinson, Palo Alto, CA |  |  |
| CD64 | X54-5/7.1 | mouse | BV711 | BioLegend, San Diego, CA, USA |  |  |
| CD24 | M1/69 | rat | AF488 | BioLegend, San Diego, CA, USA |  |  |
| CD16/32 | 2.4G2 | rat | - | Becton Dickinson, Palo Alto, CA |  |  |

*Flow cytomentric expression of Foxp3 in lung homogenates*

| CD3 | 500A2 | hamster | FITC | eBioscience, San Diego, CA, USA |  |
| --- | --- | --- | --- | --- | --- |
| CD4 | RM4-5 | rat | PerCP-Cy5.5 | eBioscience, San Diego, CA, USA |  |
| Foxp3 | FJK-16s | rat | APC | eBioscience, San Diego, CA, USA |  |
| control | R35-95 | rat | PerCP-Cy5.5 | eBioscience, San Diego, CA, USA |  |
| control | eBio299Arm | mouse | FITC | eBioscience, San Diego, CA, USA |  |
| control | MOPC-21 | rat | APC | BioLegend, San Diego, CA, USA |  |
| *Cytometric bead array for cytokine measurement* | | | | |  |
| IL-1β | B122 | rat | - | eBioscience, San Diego, CA, USA |  |
| IL-2 | JES6-1A12 | rat | - | eBioscience, San Diego, CA, USA |  |
| IL-4 | 11B11 | rat | - | eBioscience, San Diego, CA, USA |  |
| IL-5 | TRFK5 | rat | - | eBioscience, San Diego, CA, USA |  |
| IL-6 | MP5-20F3 | rat | - | eBioscience, San Diego, CA, USA |  |
| IL10 | JES5-16E3 | rat | - | eBioscience, San Diego, CA, USA |  |
| IL-12p35 | C18.2 | rat | - | eBioscience, San Diego, CA, USA |  |
| IL-13 | eBio13A | rat | - | eBioscience, San Diego, CA, USA |  |
| IL-17 | 17CK15A5 | rat | - | eBioscience, San Diego, CA, USA |  |
| IL-27p19 | 5B2 | rat | - | eBioscience, San Diego, CA, USA |  |
| GM-CSF | MP1-22E9 | rat | - | eBioscience, San Diego, CA, USA |  |
| IFN-γ | AN-18 | rat | - | eBioscience, San Diego, CA, USA |  |
| TNF-α | 1F3F3D4 | rat | - | eBioscience, San Diego, CA, USA |  |
| IL-1β | polyclonal | rabbit | biotin | eBioscience, San Diego, CA, USA |  |
| IL-2 | JES6-5H4 | rat | biotin | eBioscience, San Diego, CA, USA |  |
| IL-4 | BVD6-24G12 | rat | biotin | eBioscience, San Diego, CA, USA |  |
| IL-5 | TRFK4 | rat | biotin | eBioscience, San Diego, CA, USA |  |
| IL-6 | MP5-32C11 | rat | biotin | eBioscience, San Diego, CA, USA |  |
| IL10 | JES5-2A5 | rat | biotin | eBioscience, San Diego, CA, USA |  |
| IL-12p40 | C17.8 | rat | biotin | eBioscience, San Diego, CA, USA |  |
| IL-13 | eBio1316H | rat | biotin | eBioscience, San Diego, CA, USA |  |
| IL-17 | eBio17B7 | rat | biotin | eBioscience, San Diego, CA, USA |  |
| GM-CSF | MP1-31G6 | rat | biotin | eBioscience, San Diego, CA, USA |  |
| IFN-γ | R4-6A2 | rat | biotin | eBioscience, San Diego, CA, USA |  |
| TNF-α | XT3/XT22 | rat | biotin | eBioscience, San Diego, CA, USA |  |
| Streptavidin |  |  | PE | eBioscience, San Diego, CA, USA | |

Abbreviations: AF, Alexa Fluor; APC, allophycocyanine; BV, Brilliant Violet; Cy, cyanine; FITC, fluorescein isothiocyanate; PerCP, peridinin chlorophyll protein; PE, phycoerythrin.
