# Supplementary material for: Prevention of allergy by virus‐like nanoparticles (VNP) delivering shielded versions of major allergens in a humanized murine allergy model
Source: Allergy. 2018 Nov 5;74(2):246–60. doi: 10.1111/all.13573 (PMC6587790; doi:10.1111/all.13573)
Supplement: Supplementary file 9 [file ALL-74-246-s009.docx]

**Table S2. Comparison of allergen-specific T cell proliferation induced by different concentrations of MA::Art v 1 and Art v 1::GPI VNP by [3H]-thymidine uptake**

| **Total protein concentration**  **[µg/ml]** | **Mean±SEM VNP**  **MA::Art v 1**  **[cpm]** | **Mean±SEM VNP**  **Art v 1::GPI**  **[cpm]** | **p-value*** | **Significance level** |
| --- | --- | --- | --- | --- |
| 10.000 | 76202±7587 | 90061±15728 | 0.4710 | ns |
| 3.333 | 54246±5277 | 37733±4124 | 0.0170 | * |
| 1.111 | 37667±3421 | 21270±2630 | 0.0004 | *** |
| 0.370 | 24038±2821 | 8159±809 | <0.00001 | *** |
| 0.123 | 12430±1715 | 2700±253 | <0.00001 | *** |
| 0.041 | 4272±671 | 1387±167 | 0.0001 | *** |
| 0.014 | 1547±206 | 889±53 | 0.0032 | ** |
| 0.004 | 1002±97 | 885±55 | 0.3005 | ns |

*) p-values show comparisons between MA::Art v 1- and Art v 1::GPI-induced proliferation at the indicated concentrations. ns, not significant; *, p<0.05; **, p<0.01; ***, p<0.001. cpm, counts per minute.
