# Supplementary material for: Prevention of allergy by virus‐like nanoparticles (VNP) delivering shielded versions of major allergens in a humanized murine allergy model
Source: Allergy. 2018 Nov 5;74(2):246–60. doi: 10.1111/all.13573 (PMC6587790; doi:10.1111/all.13573)
Supplement: Supplementary file 10 [file ALL-74-246-s010.docx]

**Table S3A. β-hexosaminidase release induced by MA::Art v 1 VNP**

| **Total protein concentration**  **[µg/ml]** | **Art v 1 concentration**  **[µg/ml]** | **Mean±SEM β-hexosaminidase release**  **[%]** |
| --- | --- | --- |
| 100 | 35.8 | 13.93±1.36 |
| 10 | 3.6 | 4.89±0.486 |
| 1 | 0.4 | 4.25±0.352 |
| 0.1 | 0.04 | 3.51±0.10 |

**Table S3B. β-hexosaminidase release induced by Art v 1::GPI VNP**

| **Total protein concentration**  **[µg/ml]** | **Art v 1 concentration**  **[µg/ml]** | **Mean±SEM β-hexosaminidase release**  **[%]** |
| --- | --- | --- |
| 100 | 17.8 | 58.23±9.52 |
| 10 | 1.8 | 36.63±3.27 |
| 1 | 0.2 | 9.43±0.94 |
| 0.1 | 0.02 | 3.92±0.23 |

**Table S3C. β-hexosaminidase release induced by rArt v 1**

| **Total protein concentration**  **[µg/ml]** | **Art v 1 concentration**  **[µg/ml]** | **Mean±SEM β-hexosaminidase release**  **[%]** |
| --- | --- | --- |
| 10 | 10.00 | 36.17±6.31 |
| 1 | 1.00 | 41.18±9.19 |
| 0.1 | 0.10 | 36.30±7.67 |
| 0.01 | 0.01 | 18.69±1.96 |

Table shows the β-hexosaminidase release of RBL-2H3 cells sensitized with pooled sera of mugwort-sensitized mice induced by the indicated VNP or rArt v 1. Release with control particles without antigen: 7.2±0.92%
