# Supplementary material for: Prevention of allergy by virus‐like nanoparticles (VNP) delivering shielded versions of major allergens in a humanized murine allergy model
Source: Allergy. 2018 Nov 5;74(2):246–60. doi: 10.1111/all.13573 (PMC6587790; doi:10.1111/all.13573)
Supplement: Supplementary file 11 [file ALL-74-246-s011.docx]

**SUPPLEMENTAL INFORMATION FOR ONLINE SUPPLEMENT-UNMARKED**

**Prevention of allergy by virus-like nanoparticles (VNP) delivering shielded versions of major allergens in a humanized murine allergy model**

#### Running title: Shielding of allergens inside virus-like nanoparticles.

Bernhard Kratzer MSc^1^, Cordula Köhler BSc^1^, Sandra Hofer BSc^1^, Ursula Smole PhD^1^, Doris Trapin MSc^1^, Jagoba Iturri PhD^2^, Dietmar Pum PhD^2^, Philip Kienzl MD^3^, Adelheid Elbe-Bürger PhD^3^, Pia Gattinger MSc^4^, Irene Mittermann PhD^4^, Birgit Linhart PhD^4^, Gabriele Gadermaier^5^, Beatrice Jahn-Schmid^4^, Alina Neunkirchner, PhD^1^, Rudolf Valenta MD^4^ and Winfried F. Pickl MD^1^

^1^Institute of Immunology, Center for Pathophysiology, Infectiology and Immunology, Medical University of Vienna, Vienna, Austria

^2^Department of Nanobiotechnology, Institute for Biophysics, University of Natural Resources and Life Sciences Vienna, Vienna, Austria

^3^Department of Dermatology, Division of Immunology, Allergy and Infectious Diseases, Medical University of Vienna, Vienna, Austria

^4^Institute of Pathophysiology and Allergy Research, Center for Pathophysiology, Infectiology and Immunology, Medical University of Vienna, Vienna, Austria

^5^Division of Allergy and Immunology, Department of Biosciences, University of Salzburg, Salzburg, Austria

**Corresponding author:**

Winfried F. Pickl, MD

Institute of Immunology, Center for Pathophysiology, Infectiology and Immunology, Medical University of Vienna, Lazarettgasse 19, 1090 Vienna, Austria.

Phone: (+431) 40160 33245.

Fax: (+431) 40160 933245.

Email: [winfried.pickl@meduniwien.ac.at](mailto:winfried.pickl@meduniwien.ac.at).

ORCID ID: orcid.org/0000-0003-0430-4952

**MATERIALS AND METHODS**

**Cell lines**

A lab isolate of the HEK-293T cell line (human embryonic kidney epithelial cells) was maintained in IMDM (GE Healthcare, Pasching, Austria) plus 10% FCS (Invitrogen, Carlsbad, CA) RBL-2H3 cells were cultured in RPMI 1640 (GE healthcare) plus 10% FCS.

**Transfection of HEK-293T cells for monitoring surface and intracellular expression of allergen fusion proteins**

The day before transfection, 1 x 10^6^ HEK-293T cells were seeded into 100 mm cell culture dishes (Sarstedt, Nürnbrecht, Germany). Two hours before transfection medium was changed. Transfection was performed with 30 µg of the following pEAK12 expression plasmids, MA::Art v 1, MA::Bet v 1, Art v 1::GPI, Bet v 1::GPI, GFP. Empty pEAK12 plasmid was used as negative control. After 18 hours medium was changed and cells were cultured for another 24 hours.

**Plasmid constructs**

For the construction of MA::Art v 1, the full-length human codon optimized Art v 1 gene was amplified from pCI::hArt v 1(1) (kindly provided by Josef Thalhamer, University of Salzburg, Austria) using the following primers (restriction enzyme recognition sites underlined): MA_for: 5’-GCGGGGGCTAGCatggccggcagcaagctgtgcgaga and MA_rev: 5’- GCTCGAAGCGGCCGCCCGTctaga. The *Nhe I* and *Not I* digested Art v 1 gene was subcloned into the *Nhe I* and *Not I* digested pEAK12::MA::GFP (2) vector replacing GFP. Similarly, Art v 1::GPI was constructed by amplifying Art v 1 gene from pCI::hArt v 1 using the the primers GPI_for: 5’- gcgggaagcttGCCTCGAGATGGATGCAATGAAGAG and GPI_rev: 5’- GCCCGTctagaGGCgtgggtgc. The Hind III and Xba I digested Art v 1 was subcloned into the Hind III and Nhe I digested pEAK12::mCD80::CD16bGPI (3) expression vector releasing mCD80.

For the construction of MA: Bet v 1, the full-length human codon optimized Bet v 1 gene was amplified from Bet v 1-α-crystallin (kindly provided by Barbara Bohle, Medical University of Vienna, Austria) using the following primers (restriction enzyme recognition sites underlined): MA_for: 5’-GCGGGGCTAGCGGTGTTTTCAATTACGAAACTGAGAC and MA_rev: 5’-gggcggcggccgctttagttgtaggcatcggagtgtg. The *Nhe I* and *Not I* digested Bet v 1 gene was subcloned into the *Nhe I* and *Not I* digested pEAK12::MA::GFP (2) vector replacing GFP. Similarly, Bet v 1::GPI was constructed by amplifying Bet v 1 gene from Bet v 1-α-crystallin using the the primers GPI_for: 5’- cgcgggagatctcggtgttttcaattacgaaactgagacc and GPI_rev: 5’-gcgcccgctagcgccgccgccgttgtaggcatcggagtgtgcc. The Bgl II and Nhe I digested Bet v 1 was subcloned into the Bam H I and Nhe I digested pEAK12::mCD80::CD16bGPI (3) expression vector releasing mCD80. The proper sequence of the constructs was verified by DNA sequencing (Eurofins genomics, Ebersberg, Germany). The original MoMLV *gag*-*pol* (OGP) was expressed using the mammalian expression vector pMD.gagpol kindly provided by Dr. R. Mulligan, Boston, MA.

**Immunofluorescence analyses of producer cell lines**

For membrane staining of Art v 1, 5 x 10^5^ HEK-293T cells were incubated at 4°C with the Art v 1-specific mAb clone 5 (1) or isotype control mAb for 30 minutes. Afterwards, cells were washed with 4.5 ml of PBS containing 0.5% BSA, 0.05% NaN_3_ and 20 µl of appropriately diluted secondary anti-mouse antibody was added. Cells were incubated at 4°C for 30 minutes, washed as above and analyzed on a BD FACS Calibur flow cytometer equipped with the CellQuest software (Becton Dickinson, Franklin Lakes, NJ). (4) For intracellular staining of HEK-293T cells, 5-8 x 10^6^ cells were fixed in 70% ice-cold ethanol in ddH_2_O and incubated for 48-72 hours. Subsequently, cells were washed twice with 15 ml of PBS containing 0.5% BSA and 0.1% Triton X-100. Aliquots of 5 x 10^5^ cells were stained and analyzed as described above.

**Biochemical analyses of producer cells and VNP**

Whole cell lysates of HEK-293T cells were obtained by incubating 1 x 10^6^ cells in 10 µl lysis buffer containing 1% NP-40, 1 mM EDTA, 1 mM PMSF, 20 µg/ml aprotinin and 20 µg/ml leupeptin (Biorad, Hercules, CA) in TBS (50 mM Tris, 150 mM NaCl) on ice for one hour. Subsequently, lysates were centrifuged at 80 g in a microfuge (Eppendorf, Hamburg, Germany) to remove insoluble material at 4˚C for 10 minutes and mixed with 4 x Laemmli sample buffer as described previously.(5) Similarly, purified VNP were mixed with 4 x Laemmli sample buffer. Cellular and VNP lysates were resolved by 4-20% SDS-PAGE (BioRAD). Subsequently, proteins were transferred onto nitrocellulose membranes (Biorad) followed by incubation with MoMLV p30Gag-specific mAb R187 (ATCC, Manassas, VA) anti-Art v 1 mAb clone 5,(1) or 1:10 diluted serum of mice, which had been exposed to VNP or mugwort pollen extract as indicated. For the detection of primary antibody binding, a HRP conjugated anti-mouse Ig secondary reagent (DAKO, Glostrup, Denmark) was used at a dilution of 1:10^4^. After extensive washing, blots were developed with a luminol-based indicator system (Biorad) and photographs were taken with the chemiluminescent imaging system LAS-4000 (GE Healthcare).

**Lipid Raft analyses**

The analysis of lipid raft targeting of molecules of interest was performed as described previously. Briefly, 1 x 10^8^ transfected HEK-293T cells were washed in PBS and lysed in 1% Triton X-100 (Sigma)-containing buffer. Cells lysates were Dounce-homogenized and cleared from particulate material by centrifugation at 4°C and 100 x g for 10 minutes. Subsequently, cleared lysates were mixed with an equal volume of 80% sucrose (weight/volume in MES-buffered saline, pH 6.5 containing 25 mM MES, 150 mM NaCl, Sigma) and placed in a SW55 centrifuge tube (Beckmann), overlaid with 2 ml of 30% sucrose followed by 1 ml of 5% sucrose and spun at 200,000 g and 4°C for 16 to 18 hours. Nine fractions (0.5 ml each) were collected after centrifugation from the top to the bottom of gradients. Detergent-insoluble proteins were typically located in top fractions #2 and #3, which become visible at the 5 to 30%-interface, while in bottom fractions #6 to #9 of the gradients soluble cellular proteins become concentrated. Equal aliquots of different fractions were mixed with 4 x Laemmli sample buffer and resolved by 4-20% SDS-PAGE (Biorad). Subsequently, proteins were blotted onto nitrocellulose membranes (Biorad) and subjected to immunoblotting using anti-Art v 1, CD59, or CD147 mAb as listed in **Table S2**. Blots were developed as described above.

In addition, the method described by Schatzlmeier et al.(6) was used to confirm lipid raft targeting of molecules of interest in HEK 293T cells. Briefly, 1 x 10^6^ transfected HEK 293T cells were washed in PBS and overlaid onto an iodixanol gradient (Sigma; top to the bottom: 0%, 5%, 10%, 25% and 35%, respectively) containing 0.5% NP-40 within the 10% iodixanol fraction. Gradients were centrifuged at 1,000 g at 4°C for 10 minutes to pass the cells through the different layers. Subsequently, the detergent soluble and resistant membrane fractions were collected at the interfaces between the 5 - 10% and the 25 -35% iodixanol layers, respectively. Subsequently, 10 µl of the detergent resistant membranes or 25 µl of the detergent soluble membranes were mixed with 4x Laemmli loading buffer, resolved by 4-20% SDS-PAGE (Biorad) and transferred onto nitrocellulose membranes (Biorad) and immunoblotted as described above.

**Dynamic light scattering and zeta potential characteristics of VNP**

DLS and zeta potential determinations were conducted with VNP resuspended in PBS at a protein concentration of 0.1 µg/ml on a Zetasizer Nano-ZS apparatus (Malvern, Herrenberg, Germany) equipped with a 633 nm laser line at room temperature. Mean values and standard deviations of count rate and number weighted diameters were calculated from six independent measurements for each sample.

**Negative stain electron microscopy**

Ultrastructural characterization of VNP was performed by transmission electron microscopy on a Tecnai T20 microscope (FEI, Eindhoven, The Netherlands) at 160 kV. For negative staining, VNPs were adsorbed onto 300-mesh carbon coated copper grids (Groepl Elektronenmikroskopie, Tulln, Austria) and negatively stained in 1% uranyl acetate for 3-5 minutes (Sigma).

**Generation of bone marrow-derived dendritic cells (BMDC)**

Bone marrow-derived dendritic cells were prepared according to standard protocols as described previously.(3) Briefly, femurs of sacrificed TCR/DR1 transgenic mice were obtained after anatomical dissection. BM cells were flushed out of the prepared femurs and resuspended at a density of 2 x 10^5^ cells/ml in RPMI 1640 (GE Healthcare), 10% FBS (Gibco, Waltham, MA), gentamycin (Gibco), 20 ng/ml GM-CSF (Preprotech, London, UK). Medium was replaced every other day and the free floating fraction of cultures encompassing fully differentiated BMDCs were harvested after 10 days. For the assessment of DC-activation, 2 x 10^5^ BMDC were incubated in 96 well plates (Sarstedt) in a total volume of 200 µl with either 10 µg/ml VNP, 100 µg/ml aqueous mugwort pollen extract (Greer, Lenoir, NC), 5 µg/ml rArt v 1 (Biomay AG, Vienna, Austria) or 100 ng/ml LPS (Invivogen, San Diego, CA) for 24 hours. Alternatively, BMDC initially incubated as above were further co-incubated with 100 µg/ml aqueous mugwort pollen extract, 100 ng/ml LPS or medium alone for another 24 hours. From both types of cultures 130 µl of supernatants were removed and subjected to cytokine analyses by multiplexing (Luminex-100, Merck-Millipore). BMDC were harvested and the expression on the fully differentiated CD11c^+^CD11b^+^ BMDC of CD80 (clone identity and conjugation in **Table S2**), CD86, CD40 and MHC II (HLA-DR1) molecules was assessed by flow cytometry on a LSR Fortessa flow cytometer (Becton Dickinson) and analyzed with the FlowJo 10.2 software package (FlowJo LCC, Ashland, OR). (4)

**Cellular proliferation assays**

Spleens were isolated from TCR/DR1 mice according to standard protocols. Briefly, spleens were homogenized in RPMI 1640 plus 10 % FCS, 2 mM L-glutamine, essential amino acids, 0.1 mM 2-mercaptoethanol, 1 mM sodium pyruvate and 25 mM HEPES (pH 7.3) medium. A single cell suspension was obtained by mincing organs through a 70 µm cell strainer (Becton Dickinson). Erythrocytes were removed by incubation in ammonium chloride lysis buffer containing 155 mM ammonium chloride (Merck, Darmstadt, Germany), 10 mM potassium hydrogen carbonate (Merck), 0.1 mM EDTA (Sigma-Aldrich, St. Louis, MO, pH 7.40) at room temperature for 5 minutes. Single cell suspensions of splenocytes (2 x 10^5^/well) were incubated in 96-well round bottom plates (Sarstedt) with the indicated stimuli. After 72 hours, cells were pulsed with methyl-[3H]thymidine (1 µCi/well) for 18 hours and T cell proliferation was quantified on a Betaplate Counter (Perkin Elmer, Waltham, MA).

**VNP binding and uptake studies**

Single cell suspensions of splenocytes (1 x 10^6^) were incubated with red-fluorescent (mCherry) VNP (100 µg total protein) in 120 µl at 4°C or 37°C in 5 ml polystyrene tubes (Becton Dickinson) for 1 hour. After incubation, cells were washed twice and stained with a panel of mAbs as listed in **Table S2** at 4°C for 30 minutes. Samples were acquired on a LSR Fortessa flow cytometer (Becton Dickinson) and analyzed with the FlowJo 10.2 software package (FlowJo LCC, Ashland, OR).

**RBL-Assay**

RBL assay was performed as described previously.(7) Briefly, one day before the assay, RBL-cells were trypsinized, washed with RPMI+10% FBS and resuspended and 60.000 cells were seeded into each well of a 96 well flat bottom plate (Sarstedt). After 16-18h of incubation at 37°C plus 5% CO_2_ 3µl of Art-v 1 specific IgE containing mouse serum was added to each well to sensitize RBL cells for 2 h. After this incubation time, cells were washed twice with 200 µl of tyrodes buffer (Sigma Aldrich) and 100 µl tyrodes buffer containing the respective stimulus were added to each well and incubated for 1 h at 37°C plus 5% CO_2_. Final concentrations of rArt v 1 (Biomay AG, Vienna, Austria) in the assay were 10, 1, 0.1 and 0.01 µg/ml, respectively. Final concentrations of VNP (total protein) were 100, 10, 1 and 0.1 µg/ml. Art v 1-specific protein concentrations within the two different VNP preparations were determined by a competitive ELISA assay and were plotted against the β-hexosaminidase release (Fig 4A and B). The cross-linking anti-mouse IgE mAb R35-72 (BD-Pharmingen) was used at a fixed concentration of 10 µg/ml as FcεRI/IgE-specific positive control. For 100% release, cells were lysed with 10% Triton X-100. Subsequently, cells were centrifuged at 500 x g for 5 min, 50µl of the b-hexosoaminidase containing supernatant was taken of, transferred to a new 96 well flat bottom plate (Brand) and mixed with 50 µl of substrate solution (0.1 M Citric Acid, 16 µM 4-MUG, pH 4.5), incubated for 1h at 37°C and stopped with 100 µl of 0.2 M Glycine, pH 10.7. Fluorescence signal (excitation 360nm, emission 465 nm) was measured on a Mithras LB 940 microplate reader (Berthold Technologies, Bad Wildbad, Germany).

In addition, huRBL assays were performed as described previously (8) In brief, human sera or plasma (diluted 1:10 in RPMI 1640 medium plus 10% FCS) of mugwort allergic individuals were incubated with RBL-ATL8, a rat basophilic leukemia (RBL) cell line expressing the human FcεRI α/β/γ subunits (9), overnight and were washed as described above. Degranulation of sensitized RBL-ATL8 cells was induced by adding rArt v 1, VNP expressing either the shielded or surface exposed Art v 1 in equimolar concentrations of Art v 1 or alternatively empty VNP (66 µg/ml). The cross-linking anti-human IgE mAb E124.2.8 (Beckman Coulter, Brea, CA) was used at a fixed concentration of 200 µg/ml as hFcεRI/hIgE-specific positive control. The percentages of beta-hexosaminidase release were calculated as above.

**Patients’ sera**

Sera from 5 mugwort pollen-allergic patients were included who all had recurrent rhinitis/conjunctivitis during late summer, positive skin prick tests and specific IgE (RAST >3) to mugwort pollen (ImmunoCAP, Thermo Fischer Scientific, Phadia, Uppsala, Sweden), approved by the ethical commission of the Medical University of Vienna (EK No. 497/2005). All patients were sensitized to Art v 1 as determined by IgE-immunoblots and ELISA.

**Analyses of lung homogenates**

Lungs were excised, chopped into 10 mm^3^ pieces and incubated with RPMI1640 containing liberase TL (0.05 mg/ml; Sigma Aldrich) at 37°C for 45-60 minutes. Subsequently, cells were disaggregated by passing through a 70 µm nylon cell strainer (BD). Single cell suspensions were collected by centrifugation at 500 x g for 5 minutes. Cell pellets were re-suspended in ammonium-chloride lysis buffer, and cell suspensions incubated at room temperature for 3 minutes, washed once in 1 x RPMI1640 plus 10% FBS before they were subjected to flow cytometric or restimulation assays. For restimulation assays, 2.5 x 10^5^ cells/well were co-incubated in 96-well flat bottom plates with either 1 µM of Art v 1_23-36_ peptide (Proimmune, Oxford, UK), 100 µg/ml aqueous mugwort pollen extract (Greer), PHA (Sigma) or medium alone. After 72 hours, plates were centrifuged at 1,000 g for 10 minutes and 130 µl of supernatant was preserved for cytokine analyses.

For staining of nuclear Foxp3, 1 x 10^6^ cells from lung homogenates were fixed with the Foxp3 transcription factor staining buffer set (Thermo-Fischer) according to the manufacturer's recommendations for 1 hour and subsequently washed 2 x with washing buffer (PBS, 0.5% BSA and 0.05 % NaN_3_). Afterwards, cells were stained with CD3, CD4 and Foxp3 mAbs as specified in **Table S2**. Briefly, cells were permeabilized by washing twice in permeabilization buffer, stained with the indicated antibodies for 30 minutes and subsequently washed with washing buffer. Cells were acquired on a LSR Fortessa flow cytometer (Becton Dickinson) and analyzed with the FlowJo 10.2 software package (FlowJo LCC, Ashland, OR). (4)

**Enzyme linked immunosorbent assay (ELISA)**

*Competitive ELISA for the determination of allergen content in VNP preparations.*

To determine the amount of Art v 1 within the two different VNP preparations, an Art v 1 specific monoclonal antibody recognizing the defensin domain was used. (10) For the ELISA itself, 40 µl of rArt v 1 (2 µg/ml) in bicarbonate buffer were coated overnight onto 96-well half area ELISA plates (Greiner, Kremsmünster, Austria). Subsequently plates were washed three times with washing buffer (PBS, 0.05% Tween 20) and blocked at room temperature with PBS, 0.05% Tween 20 and 1% BSA for two hours. Defined titrated amounts (total protein) of the different VNP preparations were lysed with TTX-100 and incubated with defined amounts of the Art v 1-specific mAb for 2 hours at 37°C. Free antibodies were determined by binding to plate-bound antigen (i.e., rArt v 1), using rArt v 1 as the standard for calibrating the ELISA for two hours at 37°C. Plate-bound antibodies were detected by an anti-mouse Ig-HRP antibody (DAKO, Glostrup, Denmark), washed five times, developed with TMB substrate (Sigma-Merck) for 1-2 minutes after which the reaction was stopped with 2 M HCl (Thermo). The optical density at 450 nm was measured on a Multiskan GO apparatus equipped with the SkanIt software (Thermo Fisher). Art v 1 protein concentrations were determined by comparison with the standard curve obtained with rArt v 1.

*Direct ELISA for the determination of Art v 1 or VNP-specific immunoglobulins.*

For that purpose, 100 µl of rArt v 1 (2 µg/ml) or 10 µg/ml of empty VNP in bicarbonate buffer, were coated onto 96-well ELISA plates (NUNC maxisorp, Sigma-Merck) overnight. Subsequently, plates were washed three times with washing buffer (PBS, 0.05% Tween 20) and blocked with PBS, 0.05% Tween 20, 1% BSA for 2 hours. Diluted serum samples (IgE, 1:15; IgG1, 1:1000; IgG2a, 1:500) were applied in duplicate and incubated overnight. Then, plates were washed five times, incubated with the respective rat-anti-mouse isotype specific antibodies (Becton Dickinson, 1:500) for 3 hours and washed five times. Finally, plates were incubated with HRP-conjugated goat-anti-rat Ig (GE Healthcare) at 37°C and 4°C, 30 minutes each, washed 5 x and developed with 2,2′-Azino-bis(3-ethylbenzothiazoline-6-sulfonic acid) di-ammonium salt, ABTS (Sigma Aldrich) for 2 hours followed by determination of the optical density at 405 nm using as reference wavelength 490 nm on the Multiskan GO equipped with the SkanIt software (Thermo Fisher).

***In vivo* uptake studies**

MA::Art v 1 VNP preparations were adjusted to a concentration of 1µg/µl. 200 µl of the MA::Art v 1 VNP suspension was labeled with 0.2 µl of cell mask orange (Thermo Fisher) per microgram of VNP for 10 minutes on ice. After the labeling the MA::Art v 1 VNP preparations were extensively dialyzed against PBS to remove unbound dye and the specificity of the labeling process was confirmed using a cell line (not shown). For *in vivo* uptake studies, anesthetized mice were challenged intratracheally with 30 µl of labeled MA::Art v 1 VNP and sacrificed on the next day. VNP^+^ lung cell subsets were identified by flow cytometry as described using the antibody panel listed in **Table S2**.(4, 11)

**Determination of cytokines in cell culture supernatants**

Cytokines secreted upon stimulation of splenocytes (2 x 10^5^ cells/200 µl), lung cell suspensions (2.5 x 10^5^ cells/200 µl) or BMDC (2 x 10^5^ cells/200 µl) cultured for 72 hours (splenocytes and lung cell suspensions) or 24 and 48 hours (BMDC), respectively, were determined by multiplexing (Luminex). Briefly, 30 µl aliquots of the respective cell culture supernatants were incubated with 5 x 10^3^ beads coated with the respective capture antibodies, (anti-mouse IL-1β, IL-2, IL-4, IL-5, IL-6, IL-10, IL-12, IL-13, IL-17A, IL-27, GM-CSF, INF-γ, and TNF-α) at 4°C overnight. After washing in MultiscreenHTS BV 1.2 µm plates (Merck-Millipore), beads were incubated with biotinylated secondary antibodies recognizing the respective cytokines at room temperature for one hour followed by further staining with streptavidin-PE (all reagents from eBioscience, Thermo Fisher). Fluorescence intensities of individual bead populations were related to standard curves obtained using known cytokine concentrations and absolute concentrations were calculated accordingly.

**FIGURE LEGENDS ONLINE SUPPLEMENT**

**Figure S1. Characterization of producer cells and purified VNP. A,** IB analyses of detergent resistant (DRM) and soluble (DSM) membrane fractions of producer cells confirms the targeting of MA::Art v 1 and Art v 1::GPI to lipid rafts regions. **B,** Shown is the VNP size measured by dynamic light scattering. **C,** Shown is the stability of VNP as determined by the zeta potential of the indicated VNP preparations. No significant difference regarding the molecules targeted to VNP are observed.

**Figure S2. Lack of DC activation by allergen-expressing VNP. A-C,** Shown are flow cytometry expression data of CD40, CD80, CD86, and MHC class II (HLA DR1) on BMDC upon incubation with VNP (10µg/ml) expressing MA::Art v 1, Art v 1::GPI, empty particles, medium alone, rArt v 1 (1 µg/ml), mugwort extract (100 µg/ml) or LPS (100 ng/ml) at 24 (**A**) or 48 (**B, C**) hours, respectively. **A** and **B** shows representative data of four independent experiments performed in hexaplicates while **C** shows the summary of four independent experiments performed in hexaplicates. No significant changes were detected by One-way ANOVA followed by Tuckey’s multiple comparison testing.

**Figure S3. Up-take of VNP by distinct splenocyte populations and lack of cytokine secretion by BMDC upon co-incubation with allergen-expressing VNP**

**A,** Shown are representative two parameter contour plots of splenocytes incubated with fluorescent VNP at 4°C (right panels) or 37°C (left panels) and gated on monocytes, neutrophils and CD103^+^ dendritic cells. Numbers in gates show percent cells with bound or internalized VNP, respectively. **B,** shows the respective cytokine levels after 48 h. All comparisons were made to medium control and only significant differences are indicated. Data are representative for one out of four independent experiments performed in hexaplicates. One-way ANOVA followed by Holm’s Sidak multiple comparison.

**Figure S4. Cytokines produced by splenocytes of TCR/DR1 mice incubated with allergen-expressing VNP. A-I,** Shown are cytokine levels produced by splenocytes (2x10^5^/well) of TCR/DR1 mice incubated with allergen-expressing VNP or empty particles (10µg/ml), medium alone, rArt v 1 (0.5 µg/ml) or PMA/Ionomycin for 72 hours. IL-2 (A), IL-4 (B), IL-5 (C), IL-10 (D), IL-13 (E), IL-17A (F), GM-CSF (G) IFN-γ (H) and TNF-α (I) levels. Only significant differences compared to splenocytes stimulated with medium alone are indicated. Data show the summary of three independent experiments performed in triplicate. One-way ANOVA followed by Holm’s Sidak correction for multiple comparisons.

**Figure S5. VNP specific antibody levels of treated mice. A**, Shown are representative IBs of empty particle lysates probed with pooled sera of mice exposed to the indicated VNP or mugwort extract. p30Gag mAb was used as positive control. **B**, Shown is the specific reactivity of R187 anti-p30Gag mAb with plate-bound empty particles, along with isotype and secondary antibody controls (n=3). **C-E,** Shown are VNP-specific serum levels of IgE (C), IgG1 (D) and IgG2a (E) for the different groups of mice. Data show the summary (C-E) of 12 (except six for, empty particles) treated mice per group of two independent (except one for empty particles) experiments. Only significant differences are indicated. Kruskal-Wallis test followed by Dunn’s multiple comparison.

**Figure S6. Mouse serum IgG1 and IgG2a levels and cytokines produced by lung T cells of mice pretreated with VNP and challenged with mugwort pollen extract upon re-stimulation with Art v 1_23-36_ peptide.** **A** and **B**, Shown are Art v 1-specific serum levels (day 63) of IgG1 (A) and IgG2a (B) of TCR/DR1 mice, which were i.n. pretreated with MA::Art v 1 or Art v 1::GPI VNP, empty particles or PBS followed by challenge with mugwort extract as indicated in Fig 6A. Each symbol represents an individual mouse. **C**, Shown are cytokines produced by lung cells of mice pretreated and challenged as indicated and censored at day 63 followed by re-stimulation with aqueous mugwort pollen extract (100 µg/ml) for 72 hours. Data show the summary of 13 (except four for aqueous mugwort extract) mice per group of three (except one for aqueous mugwort extract) independent experiments. Only significant differences are indicated. One-way ANOVA followed by Holm’s Sidak multiple comparison.

**Figure S7. Increased CD3^+^CD4^+^Foxp3^+^ Treg numbers in mice pretreated with MA::Art v 1 VNP. A,** Shown is the gating strategy and **B,** the Foxp3 expression levels of CD3^+^CD4^+^ T cells obtained from mouse lungs which had been treated with MA::Art v 1, Art v 1::GPI, empty particles or PBS, respectively. Numbers within gates indicate percent positive cells. Data are representative of one experiment performed out of three.

**References**

1. Bauer R, Himly M, Dedic A, Ferreira F, Thalhamer J, Hartl A. Optimization of codon usage is required for effective genetic immunization against Art v 1, the major allergen of mugwort pollen. *Allergy* 2003;**58**(10):1003-1010.

2. Kueng HJ, Manta C, Haiderer D, Leb VM, Schmetterer KG, Neunkirchner A, et al. Fluorosomes: a convenient new reagent to detect and block multivalent and complex receptor-ligand interactions. *FASEB J* 2010;**24**(5):1572-1582.

3. Derdak SV, Kueng HJ, Leb VM, Neunkirchner A, Schmetterer KG, Bielek E, et al. Direct stimulation of T lymphocytes by immunosomes: virus-like particles decorated with T cell receptor/CD3 ligands plus costimulatory molecules. *Proc Natl Acad Sci U S A* 2006;**103**(35):13144-13149.

4. Cossarizza A, Chang HD, Radbruch A, Akdis M, Andra I, Annunziato F, et al. Guidelines for the use of flow cytometry and cell sorting in immunological studies. *Eur J Immunol* 2017;**47**(10):1584-1797.

5. Kueng HJ, Leb VM, Haiderer D, Raposo G, Thery C, Derdak SV, et al. General strategy for decoration of enveloped viruses with functionally active lipid-modified cytokines. *J Virol* 2007;**81**(16):8666-8676.

6. Schatzlmaier P, Supper V, Goschl L, Zwirzitz A, Eckerstorfer P, Ellmeier W, et al. Rapid multiplex analysis of lipid raft components with single-cell resolution. *Sci Signal* 2015;**8**(395):rs11.

7. Freidl R, Gstoettner A, Baranyi U, Swoboda I, Stolz F, Focke-Tejkl M, et al. Blocking antibodies induced by immunization with a hypoallergenic parvalbumin mutant reduce allergic symptoms in a mouse model of fish allergy. *J Allergy Clin Immunol* 2017;**139**(6):1897-1905 e1891.

8. Eckl-Dorna J, Froschl R, Lupinek C, Kiss R, Gattinger P, Marth K, et al. Intranasal administration of allergen increases specific IgE whereas intranasal omalizumab does not increase serum IgE levels-A pilot study. *Allergy* 2018;**73**(5):1003-1012.

9. Nakamura R, Uchida Y, Higuchi M, Nakamura R, Tsuge I, Urisu A, et al. A convenient and sensitive allergy test: IgE crosslinking-induced luciferase expression in cultured mast cells. *Allergy* 2010;**65**(10):1266-1273.

10. Gruber P, Gadermaier G, Bauer R, Weiss R, Wagner S, Leonard R, et al. Role of the polypeptide backbone and post-translational modifications in cross-reactivity of Art v 1, the major mugwort pollen allergen. *Biol Chem* 2009;**390**(5-6):445-451.

11. Misharin AV, Morales-Nebreda L, Mutlu GM, Budinger GR, Perlman H. Flow cytometric analysis of macrophages and dendritic cell subsets in the mouse lung. *Am J Respir Cell Mol Biol* 2013;**49**(4):503-510.
